# Insights on Glucocorticoid Receptor Activity Modulation through the Binding of Rigid Steroids

**DOI:** 10.1371/journal.pone.0013279

**Published:** 2010-10-11

**Authors:** Diego M. Presman, Lautaro D. Alvarez, Valeria Levi, Silvina Eduardo, Michelle A. Digman, Marcelo A. Martí, Adriana S. Veleiro, Gerardo Burton, Adali Pecci

**Affiliations:** 1 Departamento de Química Biológica, Facultad de Ciencias Exactas y Naturales, Universidad de Buenos Aires, Buenos Aires, Argentina; 2 Departamento de Química Orgánica/UMYMFOR-CONICET, Facultad de Ciencias Exactas y Naturales, Universidad de Buenos Aires, Buenos Aires, Argentina; 3 Laboratory for Fluorescence Dynamics, Department of Biomedical Engineering and Developmental Biology Center Optical Biology Core Facility, University of California Irvine, Irvine, California, United States of America; 4 INQUIMAE-CONICET, Facultad de Ciencias Exactas y Naturales, Universidad de Buenos Aires, Buenos Aires, Argentina; 5 IFIBYNE-CONICET, Facultad de Ciencias Exactas y Naturales, Universidad de Buenos Aires, Buenos Aires, Argentina; University of Southampton, United Kingdom

## Abstract

**Background:**

The glucocorticoid receptor (GR) is a transcription factor that regulates gene expression in a ligand-dependent fashion. This modular protein is one of the major pharmacological targets due to its involvement in both cause and treatment of many human diseases. Intense efforts have been made to get information about the molecular basis of GR activity.

**Methodology/Principal Findings:**

Here, the behavior of four GR-ligand complexes with different glucocorticoid and antiglucocorticoid properties were evaluated. The ability of GR-ligand complexes to oligomerize *in vivo* was analyzed by performing the novel *Number and Brightness* assay. Results showed that most of GR molecules form homodimers inside the nucleus upon ligand binding. Additionally, *in vitro* GR-DNA binding analyses suggest that ligand structure modulates GR-DNA interaction dynamics rather than the receptor's ability to bind DNA. On the other hand, by coimmunoprecipitation studies we evaluated the *in vivo* interaction between the transcriptional intermediary factor 2 (TIF2) coactivator and different GR-ligand complexes. No correlation was found between GR intranuclear distribution, cofactor recruitment and the homodimerization process. Finally, Molecular determinants that support the observed experimental GR LBD-ligand/TIF2 interaction were found by Molecular Dynamics simulation.

**Conclusions/Significance:**

The data presented here sustain the idea that *in vivo* GR homodimerization inside the nucleus can be achieved in a DNA-independent fashion, without ruling out a dependent pathway as well. Moreover, since at least one GR-ligand complex is able to induce homodimer formation while preventing TIF2 coactivator interaction, results suggest that these two events might be independent from each other. Finally, 21-hydroxy-6,19-epoxyprogesterone arises as a selective glucocorticoid with potential pharmacological interest. Taking into account that GR homodimerization and cofactor recruitment are considered essential steps in the receptor activation pathway, results presented here contribute to understand how specific ligands influence GR behavior.

## Introduction

The glucocorticoid receptor (GR) is a ligand-regulated transcription factor, member of the nuclear-receptor (NR) superfamily that controls gene expression linked to several processes like inflammation, stress responses, glucose homeostasis, lipid metabolism, proliferation and apoptosis development [1]. Due to GR involvement in the cause and treatment of many human diseases, it is considered one of the major pharmacological targets. Many synthetic glucocorticoid drugs, such as dexamethasone (Dex) or prednisolone, are widely used in the treatment of several immunological and inflammatory diseases [2]. However, the desired immunosupresant and anti-inflammatory effects are often compromised by severe or partially nonreversible side effects [2]–[4]. To improve glucocorticoid pharmacological profile, intense efforts have been made to obtain more information about the molecular mechanisms that underlie beneficial and unwanted glucocorticoid properties, and to design new selective compounds.

In the absence of ligand, GR is associated to the hsp90 chaperone heterocomplex and primarily localizes in the cytoplasm while the GR-ligand complex is mainly nuclear. In the nucleus, the activated GR regulates gene expression through two main modes of action [5], [6]. A direct mechanism involves GR homodimer binding to positive or negative Glucocorticoid Response Elements (GRE) located in the promoter region of target genes, leading to transcription activation or repression, respectively. On the other hand, the activated GR may also function through an indirect mechanism by interacting as a monomer with other transcriptional factors, such as NFκB or AP-1 [7]. Therefore, activated GR monomers control gene expression by modulating the transcriptional activities of those transcription factors, without direct binding to DNA. Interestingly, since both GR modes of action would be independent, it has been postulated that glucocorticoid desired consequences are associated to the indirect-transrepression mechanism, while the side effects are associated to the direct transactivation one. However, this hypothesis is currently under revision as it was demonstrated that mechanistically, distinct forms of glucocorticoid-inducible gene expression are critical to the development of anti-inflammatory effects by repressing inflammatory signaling pathways and inflammatory gene expression at multiple levels [4], [8], [9]. Thus, the design of novel GR ligands should consider a detailed evaluation of which types of GR conformations relate to which specific transcriptional responses and functional outcomes.

Like most of the NRs, the GR is a modular protein that is organized into three major domains: a poorly conserved N-terminal ligand-independent activation function-1 domain (AF-1), a highly conserved central DNA-binding domain (DBD) that recognizes specific GREs in target promoters -plus a dimerization region-, and a C-terminal ligand-binding domain (LBD) [10], [11]. The LBD contains ten á-helices that fold into a globular structure, described as a net enclosing a central hydrophobic ligand-binding pocket (LBP). According to X-ray crystallography analysis, the structural plasticity of the LBP allows the binding of ligands with quite different geometries [12]–[17]; thus, bulky groups located in different positions of the steroid, fit into the LBD without affecting its global conformation. In addition to the LBP, the LBD also contains a dimerization interface and a hydrophobic domain (AF-2), involved in the interaction with cofactors. In this way, ligand structure may influence GR conformational states that would modulate its ability to homodimerize and/or to recruit either coactivators or corepressors. In this sense, the understanding of how specific ligands influence the GR-LBD conformation could be a key start-point in the rational design of new selective glucocorticoid receptor modulators (SGRMs) [18].

Despite the fact that little is known about how ligand structure may affect GR dimerization, some reports have been focused on the study of GR-LBD/cofactor interactions. Particularly, it is well known that the p160 GR coactivators family contain multiple LxxLL motifs in which leucine residues are presented on one face of the amphipathic helix making it to be in contact with the AF-2 nonpolar groove [12]. Even though this binding site is formed by residues of helices H3, H4 and H12, its conformation is mainly determined by the H12 position. Thus, the binding of the pure agonist Dex induces a GR conformation in which the position of H12 allows the interaction with coactivators such as the transcriptional intermediary factor 2 (TIF2) [12], [19]. In contrast, H12 conformation changes when the GR binds the antagonist RU486, preventing GR-coactivator interaction [19] but allowing the recruitment of corepressors [20]. Nevertheless, interaction between TIF2 and GR-RU486 complex has been described [21], [22]. It is noteworthy that the recently obtained GR LBD-RU486 crystal structure [23] shows that H12 may adopt different positions upon RU486 binding, explaining at least in part the complex activity profile of this ligand. On the other hand, Raaijmakers et al. have recently obtained the crystal structure of the Progesterone Receptor (PR) LBD-RU486 complex in which the H12 adopts an agonistic conformation [24].

Although GR homodimerization is considered an essential step in the GR-mediated gene-activating properties, there is still a discrepancy in the identification of both, the region involved in homodimerization and the mechanisms underlying this process. In this sense, most of the evidences come from *in vitro* studies, by using the entire GR protein [25]–[30] or the GR DBD [31]–[33]. Although the DBD mutant (A458T) generates a receptor that would not be able to homodimerize *in vitro* [34], [35], several evidences suggest that LBD and AF-1 domains also participate in GR homodimerization [12], [29]. In addition, the relationship between GR/GRE interaction and the GR homodimerization process *in vivo* still is not clear [34], [36]–[40] neither is whether the coactivator recruitment occurs before, during or after GR dimer formation [41]–[44].

In previous works we studied two glucocorticoid rigid analogs, 21-hydroxy-6,19-epoxyprogesterone (21OH-6,19OP) and its 21-hemisuccinate (21HS-6,19OP). 21OH-6,19OP is a specific GR antagonist that lacks the bulky substituent at C-11 found in active antagonists of the GR [45]–[47]. The introduction of a hemisuccinate group at the 21-position of this passive antiglucocorticoid leads to a compound (21HS-6,19OP), which behaves like an agonist of GR action in direct transactivation assays [48].

Taking into account that the understanding of the molecular role played by different ligands on coactivator recruitment and dimer formation is related to the ability of predicting the overall conformational change of the receptor upon ligand binding; we had previously used Molecular Dynamics (MD) simulation to evaluate the dynamic behavior of GR LBD-Dex, GR LBD-21OH-6,19OP and GR LBD-21HS-6,19OP complexes [47], [48]. These results showed that in the receptor bound to 21OH-6,19OP the average position of the loop between helices 1 and 3 (H1–H3 loop) adopts a markedly different conformation compared to the GR LBD–Dex complex. Since according to several GR LBD crystal structures the H1–H3 loop is a fundamental region of the homodimerization interface [17], we proposed that the passive antagonist mode of action of 21OH-6,19OP would reside at least in part, in the incapacity of GR-21OH-6,19OP complex to form functional homodimers [47].

Regarding 21HS-6,19OP, those previous results led us to propose that the hemisuccinate moiety might play a key role in stabilizing the receptor active conformation of the dimerization interface, reversing the changes observed with the antagonist 21OH-6,19OP [48].

In this work, we evaluated the *in vivo, in vitro and in silico* behavior of both GR-rigid steroid complexes and we compared them with GR-Dex and GR-RU486. Using a GFPGR chimera on number and brightness (*N&B*) assays we observed that the receptor dimerizes in the nucleus independently of which ligand is bound. On the other hand, coimmunoprecipitation assays showed that coactivator recruitment of the different GR-ligand complexes depends on ligand structure, being GR LBD-21OH-6,19OP unable to recruit TIF2. Furthermore, molecular determinants that may explain the observed experimental data were found by MD simulations analyzing the interaction of GR LBD-ligand complexes and a peptide corresponding to the TIF2 coactivator. Finally, transrepression studies showed that GR-21OH-6,19OP complex inhibits NFκB and AP-1 activities. Together, these results not only give us insights on glucocorticoid receptor activity modulation but also propose 21OH-6,19OP rigid steroid as a putative novel selective glucocorticoid.

## Results

### Intranuclear distribution of GR-ligand Complexes

We began the study by analyzing the intranuclear distribution of GR-ligand complexes. Since we used the GFPGR chimera in several studies, we first evaluated the direct transcriptional activity and cellular distribution of this previously characterized fusion protein [49]. We confirmed that both agonist Dex and 21HS-6,19OP induce MMTV-driven luciferase expression in BHK cells overexpressing GFPGR, although the efficacy of the rigid steroid agonist was lower than Dex, compared with their relative efficacies reported previously [48]. On the contrary, the antagonists RU486 and 21OH-6,19OP inhibit Dex mediated GFPGR dependent gene expression (Figure S1A). Using confocal microscopy, we then tested the cellular distribution of the different GRGFP-ligand complexes in Cos-7, BHK and L929 cells. In the absence of ligand, most of the fluorescence is visualized into the cytoplasm and upon steroid addition, GR translocates to the nucleus independently of ligand structure (Figure S1B). These results indicate that the rigid steroids modulate GFPGR in a similar fashion as the wild type receptor.

**Figure 1:**
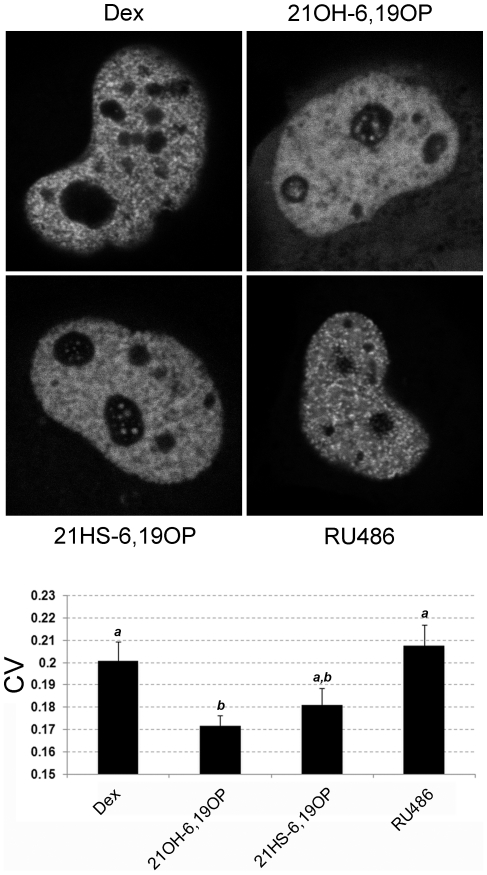
Intranuclear distribution of GFPGR. Cos-7 cells transfected with pEGFPGR were incubated with 10 nM Dex, 10 µM 21OH-6,19OP, 10 µM 21HS-6,19OP or with 1 µM RU486 for at least 40 min at 37 C. Cells were visualized by confocal scanning microscopy. Images were taken between 40 min-3 h after addition of the steroids. The figure shows representative cells for each treatment and the coefficient of variation (CV) quantitation as described in “Materials and Methods”. Bars with different superscript letters are significantly different from each other (*P*<0.05).

Analysis of the different GFPGR-complexes intranuclear distribution was performed in Cos-7 cells (Figure 1). When the rigid steroids are added, fluorescence emitted by GFPGR is randomly distributed into the nucleus at variance with GR-Dex or GR-RU486 complexes, which distribute in a punctuate manner, similar to that described previously [50]. Quantitative measurements for the randomness of the complex distribution were performed by determining the coefficient of variation number (CV) according to the method established earlier [51]. Figure 1 shows significant differences among the CVs values of 21OH-6,19OP (0.172±0.005) and Dex (0.201±0.009) or RU486 (0.208±0.009); being 21HS-6,19OP CV value in-between (0.181±0.008). According to previous works [50], our results support the idea that the nuclear distribution of GR-ligand complexes do not relate to their transcriptional activities.

### Monomeric activity of GR-ligand complexes

**Figure 2:**
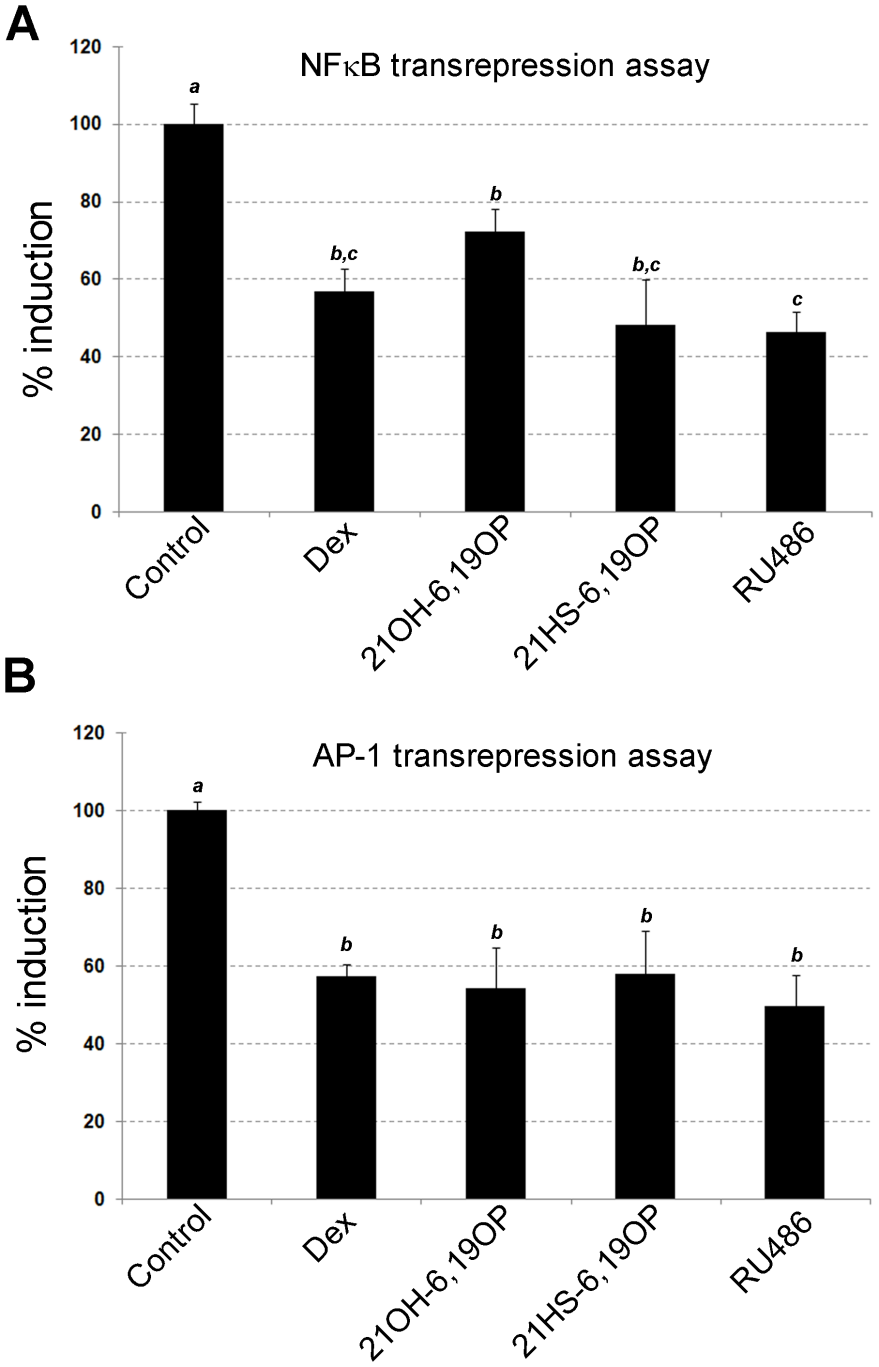
GR-ligand complexes monomeric activity. BHK21 cells were cotransfected with pkB-LUC and pRelA vectors (**A**), or pAP1-LUC and pcJun vectors (**B**). pCMV-LacZ vector were also introduced. Cells were incubated for 18 h with ethanol (Control), 10 nM Dex, 10 µM 21OH-6,19OP, 10 µM 21HS-6,19OP, and 1 µM RU486. Luciferase activity was measured. After correcting for β-galactosidase activity, values were expressed as % induction relative to the control. Means ± S.E. from three independent experiments are shown. Bars with different superscript letters are significantly different from each other (*P* <0.05).

The ability of both rigid ligands to modulate monomer-GR activities were evaluated by analyzing the complex behavior on NFκB or AP-1 mediated pathways. BHK cells were cotransfected either with pRelA expression vector and the reporter pκB-Luciferase or with pcJun and AP-1-Luciferase reporter vector, and treated with the different steroids. Figure 2 shows that, similarly to Dex and RU486, both rigid analogs are able to inhibit NFκB (Figure 2A) and AP-1 activities (Figure 2B). Therefore, although GR-21OH-6,19OP - like GR-RU486 complex - is unable to trigger a direct transcriptional response, it has the ability to act indirectly as a monomer. On the other hand, the GR-21HS-6,19OP complex, similarly to GR-Dex, exerts glucocorticoid effects for both direct transactivation (Álvarez et al. [48] and Figure S1A) and indirect transrepression (Figure 2). Moreover, considering that 21OH-6,19OP is able to transrepress both AP-1 and NFκB pathways but lacks the ability to induce transactivation (Figure S1A and also in Vicent et al. [45] and Veleiro et al. [52]); this steroid arises as a putative dissociated glucocorticoid.

### Oligomerization state of GR-ligand Complexes

Since the LBD participates actively in the contact between GR monomers [12], ligand-induced conformational changes would affect the ability of GR to form functional homodimers and consequently to induce direct transcription. We performed *in vivo* mapping of GR oligomerization state by using the *N&B* method described before [53]. This new technique, based on moment-analysis, provides the average number of moving, fluorescent molecules and their brightness at every pixel of images. In the simplest case the brightness of oligomers formed by *n* monomers are n times the brightness of monomers. Therefore, *N&B* can be used to obtain the oligomerization state of proteins in living cells with high spatial resolution [53], [54].

**Figure 3:**
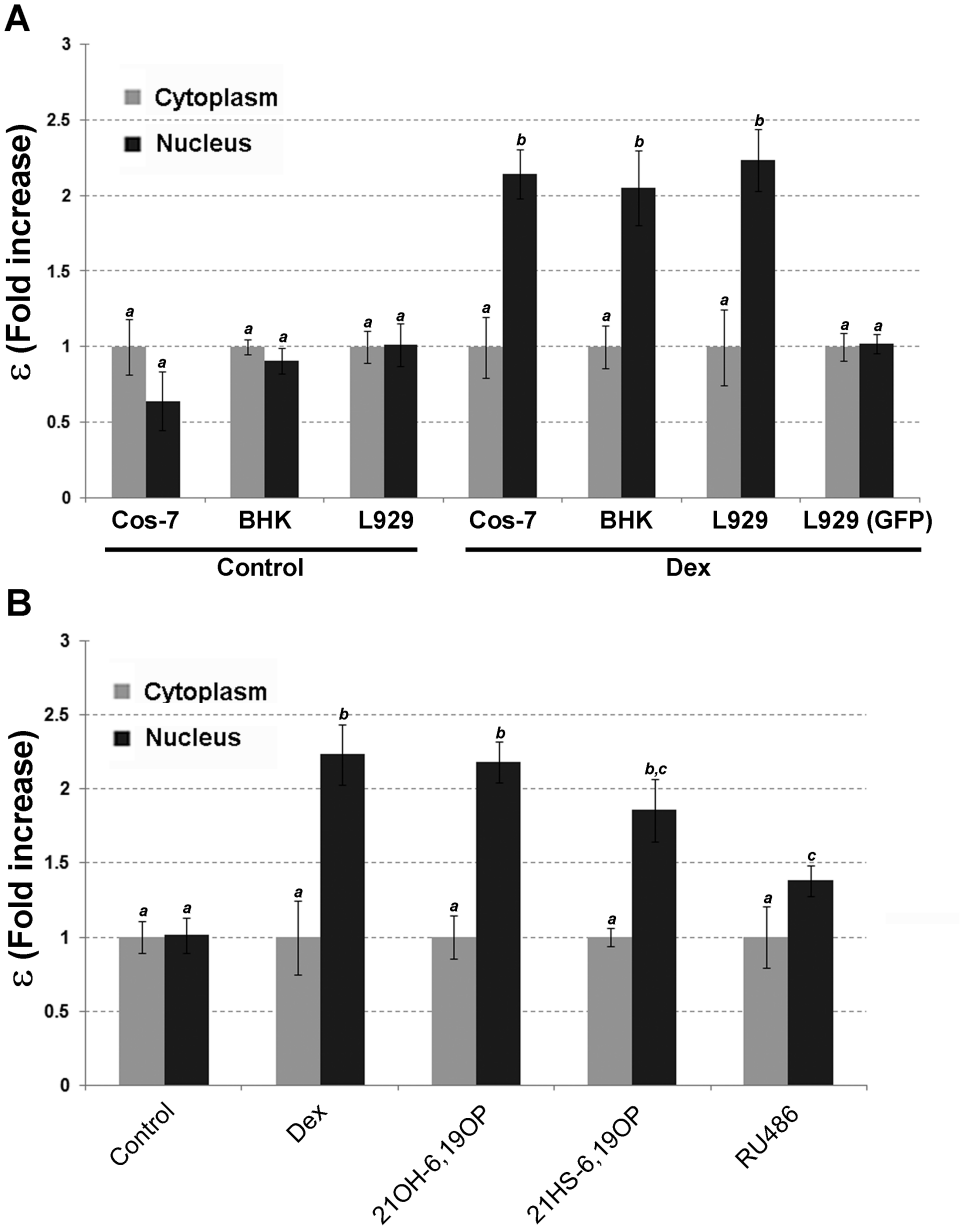
*In vivo* oligomerization analysis of GFPGR molecules. **A**. Cos-7, BHK21, and L929 cells transfected with pGFPGR were incubated with ethanol (Control) or 10 nM Dex for at least 40 min at 37 C. L929 (GFP) indicates cells tranfected with pEGFP and incubated with 10 nM Dex. **B**. L929 cells transfected with pEGFPGR were incubated with ethanol (Control), 10 nM Dex, 10 µM 21OH-6,19OP, 10 µM 21HS-6,19OP or with 1 µM RU486 for at least 40 min at 37 C. Images were taken between 40 min-6 h after steroids addition. For each cell (15≤n≤30 per treatment) the apparent brightness was calculated as described in “Materials and Methods”. The figure shows fold-increase of the real brightness (ε) relative to the cytoplasm for each cell type. Bars with different superscript letters are significantly different from each other (*P* <0.05).

Figure 3A shows the real brightness (ε) fold increase (i.e. measure of fluorophore oligomerization) corresponding to GFPGR protein expressed in different cell types. In the absence of ligand, ε values are similar in nucleus and cytoplasm; indicating the same GR oligomerization status in both cellular compartments. L929 cells transfected with GFP alone show similar absolute ε values than the unbound GFPGR (data not shown), strongly suggesting that the inactive GR is mainly a monomer. Upon Dex addition ε values significantly increase (approximately 2 fold) in the nucleus with respect to the cytoplasm in all cells analyzed (Figure 3A). These results are consistent with GR transformation and dimerization upon ligand binding. GFP ε values do not increase even in the presence of Dex, indicating the GR dependence on the oligomerization status (Figure 3A). The presence of endogenous GR molecules does not seem to interfere with the analysis, as ε values obtained in L929 or BHK cells are statistically equal to Cos-7 cells that lack endogenous GR. Consistently, it was demonstrated that transient transfection of GFPGR carrying the CMV promoter sequence inevitably results in an overexpression of GFPGR proteins [55]. Therefore, assuming that GR homodimers are the maximum oligomerization status possible for the receptor, theoretically, GR ε values should duplicate if all activated GR particles dimerize upon ligand binding. In this sense our results suggest that most of GR molecules dimerize *in vivo* after Dex treatment, although we can not empirically dismiss the possibility that higher oligomerization status may occur. Figure 3B shows a similar increase in ε values between nucleus and cytoplasm when L929 cells are treated with either of the rigid steroids. However, although RU486 treatment significantly induces receptor oligomerization, it seems to provoke less dimer formation. Together, results indicate that the activated GR particle is able to form oligomers, independently of which ligand is bound to the receptor. Moreover, results also imply that 21OH-6,19OP antagonistic effect on gene expression activation would not be related to the ability of the GR LBD-21OH-6,19OP complex to form homodimers in the nucleus.

### Dynamics of DNA-GR-ligand Complexes interaction

**Figure 4:**
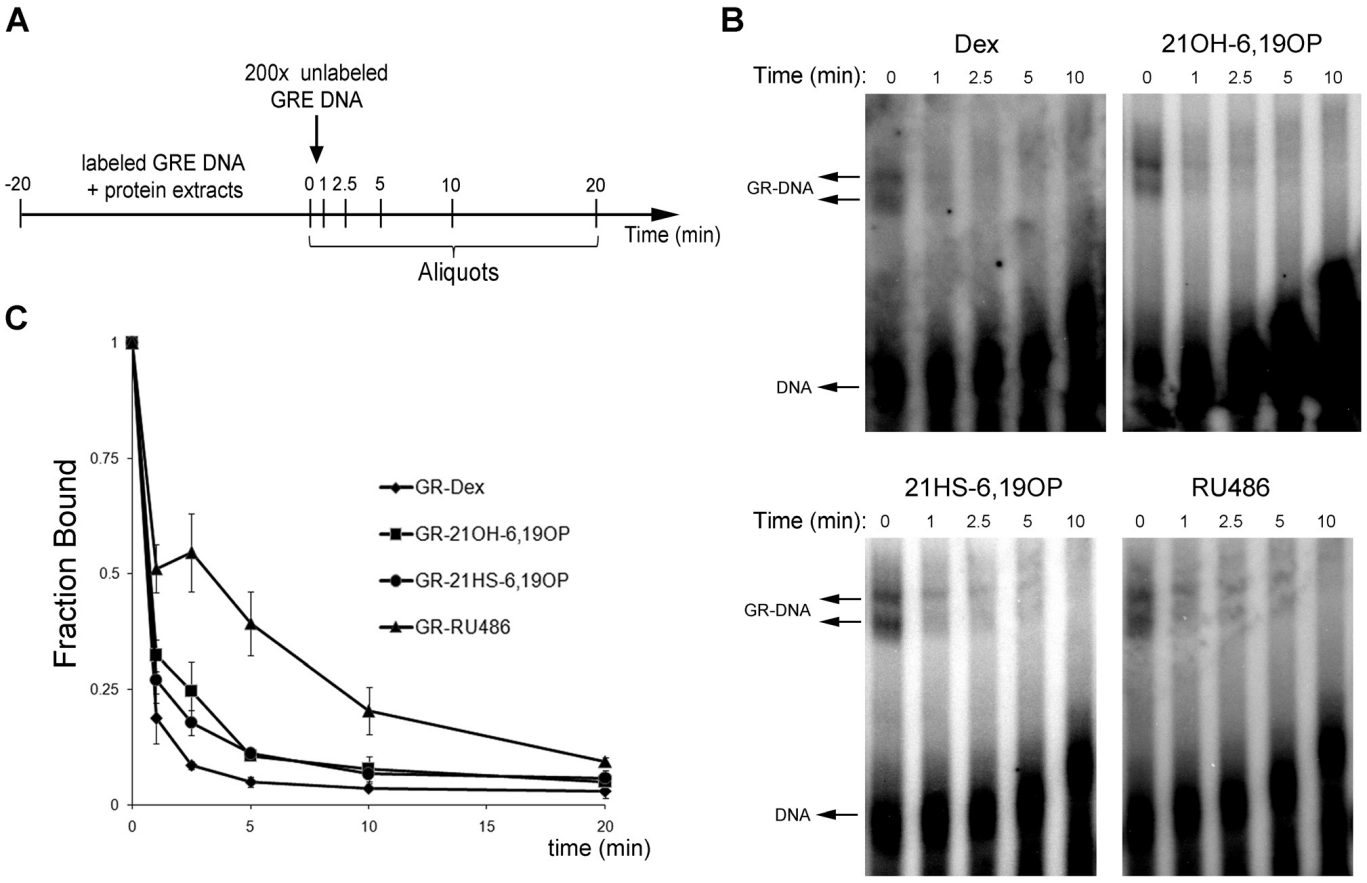
Ligand effect on *in vitro* GR-DNA dissociation. EMSAs were performed using nuclear extracts from BHK21 cells treated with ethanol (Control), 10 nM Dex, 10 µM 21OH-6,19OP, 10 µM 21HS-6,19OP and 1 µM RU486 for 30 min. Protein extracts incubated with a ^32^P-radiolabeled oligonucleotide containing a *GRE* sequence were subject to the experimental design shown in ***A*** *.* **B**. Aliquots for each treatment were loaded into a running gel and images were analyzed. **C**. Dissociation curves (mean ± S.E.) from three independent experiments show the fraction bound (relative to time zero) expressed as the ratio between GR-DNA complexes and free DNA probe. The *arrows* indicate the free DNA and the GR-DNA complexes.

To investigate the influence of ligand-induced GR conformational changes on the ability of GR to interact with specific GREs, we performed gel-shift assays with nuclear extracts obtained from BHK cells treated with different steroids. Extracts were incubated with a radiolabeled GRE-containing oligonucleotide from the MMTV promoter [28]. As it was previously reported [56]–[61], cell extracts prepared in the absence of ligand generate a double retarded band corresponding to GR-MMTV probe complexes which does not differ from ligand treated extracts (data not shown). Given this lack of ligand effects on the GR affinity for GRE-sites, we investigated whether ligand interaction affects the association/dissociation rate between GR-ligand complexes and DNA. According to previously published studies [60], a direct measurement and comparison of the association kinetics for different GR-ligand/DNA complexes is not feasible, thus we determined the dissociation kinetics. Radiolabeled probes were incubated with nuclear extracts containing GR-ligand complexes during 20 minutes, then 200× excess of specific competitor was added and reaction aliquots were loaded at different time points onto a running gel (scheme in Figure 4A). For the comparison of DNA dissociation, all extracts showed similar DNA binding at the initial time point prior to addition of excess GRE; thus, the dissociation rates were independent of the fraction of DNA bound. As already shown [60], Dex and RU486 have opposite effects on GR-DNA dissociation kinetics (Figure 4B–C). GR-Dex/DNA complex exhibits the fastest dissociation kinetics (t_1/2_ = 0.56±0.05 min) whereas the GR-RU486/DNA complex shows the slowest (t_1/2_ = 2.33±0.56 min) (Figure 4C). However, both rigid analog complexes, GR-21OH-6,19OP/DNA and GR-21HS-6,19OP/DNA have similar dissociation kinetics (t_1/2_ = 0.96±0.08 and 1.02±0.13 min., respectively) indicating that the different abilities of both rigid analogs to transactivate the MMTV promoter would not be due to the dynamic of those complexes to bind DNA.

### Cofactor interaction

**Figure 5:**
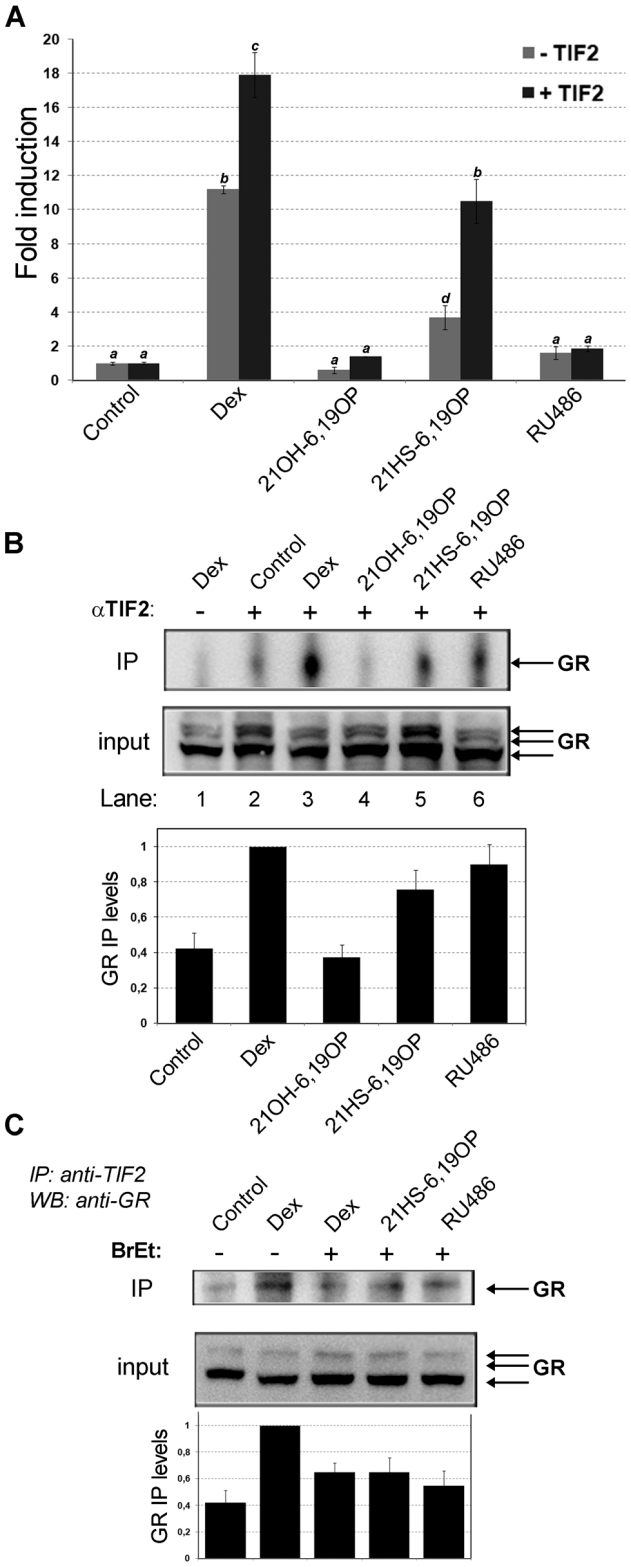
21OH-6,19OP-GR complex does not interact with TIF2. **A**. BHK21 cells were cotransfected with MMTV-Luc vector in the presence (+TIF2) or absence (-TIF2) of pTIF2 expression vector. pCMV-LacZ vector were also introduced. Cells were incubated for 18 h with ethanol (Control), 10 nM Dex, 10 µM 21OH-6,19OP, 10 µM 21HS-6,19OP, and 1 µM RU486. Luciferase activity was determined and normalized against β-galactosidase activity. Values are expressed as fold induction relative to controls. Means ± S.E. from three independent experiments are shown. Bars with different superscript letters are significantly different from each other (*P* <0.05). **B–C**. BHK21 cells cotransfected with pRSV-hGR and pTIF2 vectors were incubated with the indicated steroids for 90 min at 37 C. Cytosols were extracted and treated (or not) with ethidium bromide (BrEt) when indicated. TIF2-associated GR was analyzed as described in “Materials and Methods”. Immunoadsorptions were performed with TIF2 antibody (áTIF2). Inputs correspond to 10% of the sample previously to the immunoprecipitation protocol. Western blotting was performed with a mixture of antibodies against human GR. Arrows on input gel indicate the three isoforms of áGR (GR_A_; GR_B_; GR_C_ as previously described [21], [91]). Gels correspond to one representative experiment (n = 3). Mean ± SE values of Immunoprecipitated (IP) GR levels (relative to Dex) are shown.

Since the GR ability to induce transcription depends, at least in part, on the ligand-induced interaction with coactivators, one might postulate that the reduced transcriptional activation by a selective compound should indicate an impaired recruitment of coactivators. Previous MD simulations showed that the conformation of the AF-2 domain changes in the presence of rigid analogs [47], [48] suggesting that the binding of these steroids could affect GR ability to recruit TIF2. In order to evaluate GR/TIF2 interaction, we performed co-transfections on BHK cells with the pMMTV-Luciferase reporter in the presence or absence of pTIF2 expression vector. Therefore, we analyzed the effect of coactivator overexpression on ligand-dependent MMTV transcription activation. As expected, Figure 5A shows that TIF2 potentiates transcriptional activity of GR agonists; in fact, Dex-induced luciferase expression increases 1.60 fold when pTIF2 vector is co-transfected while 21HS-6,19OP-induced luciferase expression increases 2.85 fold. However, TIF2 overexpression is not able to generate neither RU486 nor 21OH-6,19OP agonistic activities. This result would indicate that no interaction exists between GR-RU486 or GR-21OH-6,19OP complexes and the TIF2 coactivator, or if an interaction occurs it would result in a non-functional GR-ligand/TIF2 complex.

In order to discriminate between these two options, we performed GR-ligand/TIF2 coimmunoprecipitation assays. BHK cells were co-transfected with hGR and TIF2 expression vectors and treated with different ligands. Subsequently, protein extracts were precipitated with a specific TIF2 antibody. Figure 5B shows Western blot analysis performed against GR. Results indicate that GR-21OH-6,19OP complex is not able to interact with the coactivator (Figure 5B, lane 4) while GR-Dex, GR-21HS-6,19OP and even GR-RU486 complexes bind TIF2 (Figure 5B, lanes 3, 5 and 6, respectively). Interestingly, these interactions were severely impaired in the presence of ethidium bromide throughout the precipitation reaction (Figure 5C), suggesting DNA-mediated protein association [62] between GR-ligand complexes and TIF2. Taken together, these results indicate that while 21OH-6,19OP affects GR's ability to physically interact with TIF2, the other ligands allow this interaction mainly in a DNA dependent manner. As GR-RU486 binds TIF2 without inducing MMTV transcription, we propose that this ligand would generate a non-functional complex. Thus, different mechanisms of action should be considered to explain 21OH-6,19OP and RU486 antagonism.

### Molecular dynamics simulation of GR LBD-ligand complexes

In order to investigate the molecular determinants of the Co-IP results, particularly the GR LBD-21OH-6,19OP inability to recruit TIF2, we carried out further MD simulations of GR LBD-ligand complexes bound to a peptide corresponding to the TIF2 coactivator.

**Figure 6:**
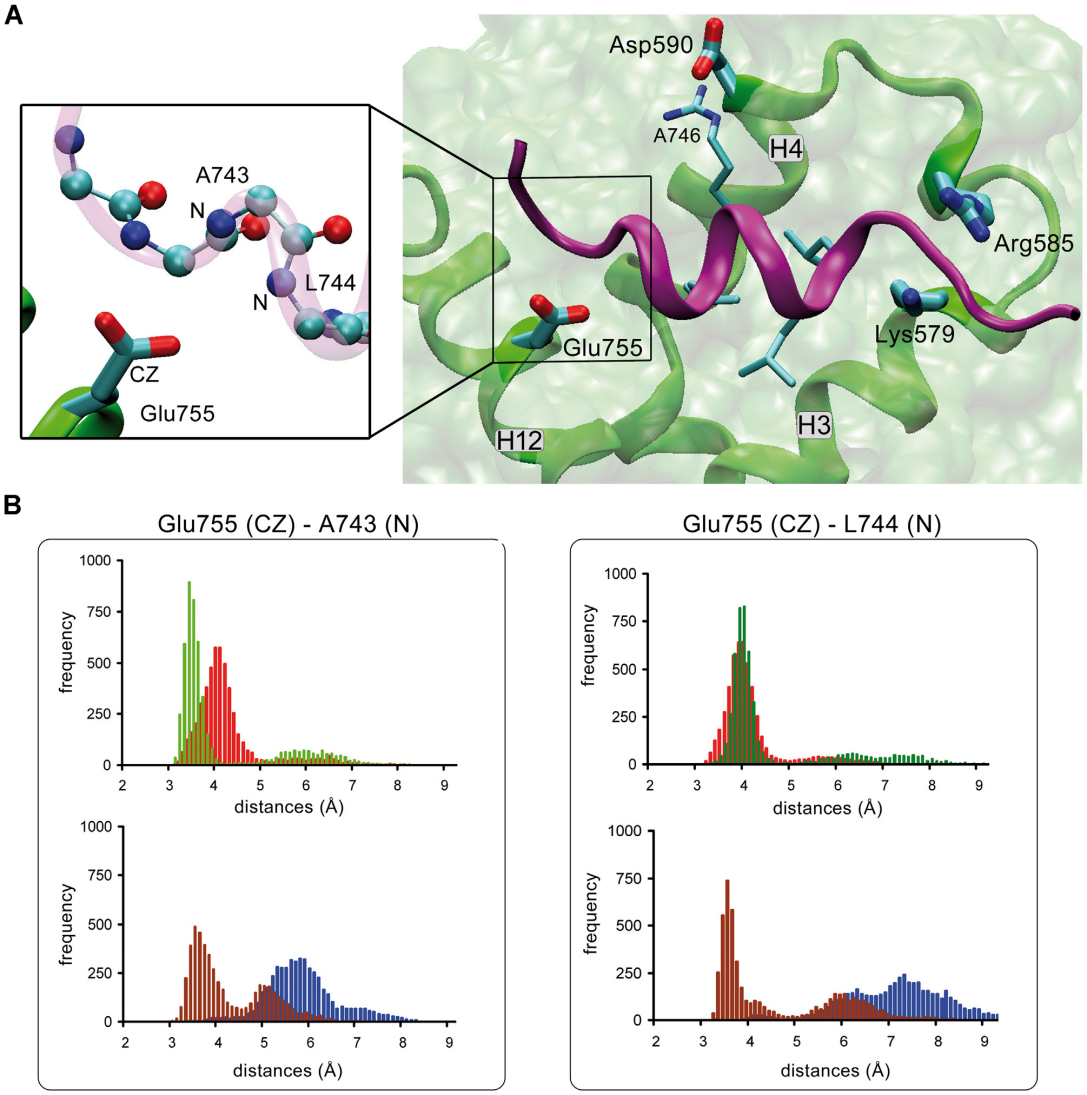
MD simulation of GR LBD-ligand/TIF2 complexes. **A**. AF-2 domain (green) is formed by helices H3, H4 and H12. GR LBD residues from Glu755, Lys579 and Arg585 have electrostatic interactions with the TIF2 coactivator backbone (violet). Asp590 residue has electrostatic interactions with the lateral side chain of TIF2 R746 residue. The figure also shows TIF2 leucines interacting hydrophobically with the GR LBD. This image is derived from the pdb:1 m2z crystal structure. **B**. Histograms of the distances between the Glu755 CZ carbon atom of GRLBD and A743 N atom or L744 N atom of TIF2 peptide. GR LBD-Dex/TIF2 (red), GR LBD-21OH-6,19OP/TIF2 (blue), GR LBD-21HS-6,19OP/TIF2 (green), GR LBD-RU486/TIF2 (brown).

The GR LBD-Dex complex (pdb:1n2z) has been crystallized together with a peptide derived from TIF2 [12]. In this structure the AF-2 domain and the TIF2 peptide interact through a hydrophobic groove conformed by GR helices H3, H4 and H12 (Figure 6A) and the leucine residues of the helical LxxLL motif. Additional electrostatic interactions termed “charge clamp”, between the GR aminoacid side chains (residues Lys579, Glu755, Arg585 and Asp590) and the peptide are also involved in GR LBD-coactivator complexes orientation and stability.

We constructed the four GR LBD-ligand/TIF2 complexes *in silico* (see “Materials and Methods”) and ran 20 ns MD simulations for each case. The trajectories of all complexes show that the global structure of the protein is reasonably stable (Figure S2A and S2B). In order to evaluate the interactions between the GR LBD and the TIF2 peptide we analyzed distances between atoms from residues Lys579, Glu755, Arg585 and Asp590 and atoms from the TIF2 peptide that in principle, may form hydrogen bond interactions (Figure 6A). We found that GR LBD Asp590 often interacts with TIF2 A746 without significant differences among the four complexes. Interestingly, according to previous results this interaction plays a key role in the specificity of the GR LBD to bind the third TIF2 LxxLL motif [12]. We also observed that Lys579 stably interacts with TIF2 backbone atoms (L748 and K751) in all GR LBD-ligand/TIF2 complexes. On the other hand, only GR-21HS-6,19OP/TIF2 complex forms a hydrogen bond between Arg585 and the TIF2 backbone atoms.

The major differences among the complexes were observed for the Glu755 and TIF2 peptide interaction (Figure 6A, amplified box). Figure 6B histogram shows that in the Dex system distances between Glu755 CZ atom and N atoms of A743 and L744 are approximately 4 Å, indicating a strong and stable interaction among these residues. In the 21HS-6,19OP system (green), while CZ (Glu755) – N (L744) distance is also around 4 Å; CZ (Glu755) – N (A743) distance is smaller, indicating a stable interaction between Glu755 and TIF2 when the agonist 21HS-6,19OP is bound. Instead, in the presence of the antagonist 21OH-6,19OP (blue) the histograms show that the distances are always higher, suggesting that Glu755 does not interact with the coactivator.

In order to study GR LBD-RU486/TIF2 complex, we first needed to evaluate GR LBD-RU486 behavior. At present, two x-ray structures of GR LBD bound to RU486 have been reported [17]; however none of them can be used as starting point for the MD simulation. In the pdb:1 nhz the helix 12 is not completely resolved [13] while in the recent x-ray structure pdb:1 h52 the GR LBD is partially unfolded [23]. Therefore, we performed the MD simulation of GR LBD-RU486 complex starting from the active conformation of the receptor (pdb:1 m2z), which also allowed direct comparison with the rest of GR-ligand/TIF2 complexes. When the RU486 molecule is introduced *in silico* into the GR LDB, atoms from the 11-substituent diethyl amino group and Leu753 (H12) side chain atoms overlap (see “Materials and Methods” and Figure S3B). However, taking into account the recently obtained PR LBD-RU486 complex crystal structure (pdb:2w8y) [24] (Figure S3A), where helix 12 is positioned in a similar way as that observed for the agonist complex GR LBD-Dex (pdb:1 m2z) [12], we introduced this ligand into the GR LBD by rotating the Leu753 side chain in order to avoid unfavorable steric interactions between the C-11 substituent in RU486 and the GR LBP atoms, obtaining in this way a GR LBD-RU486 initial complex able to be used in the MD simulation (Figure S3B).

**Figure 7:**
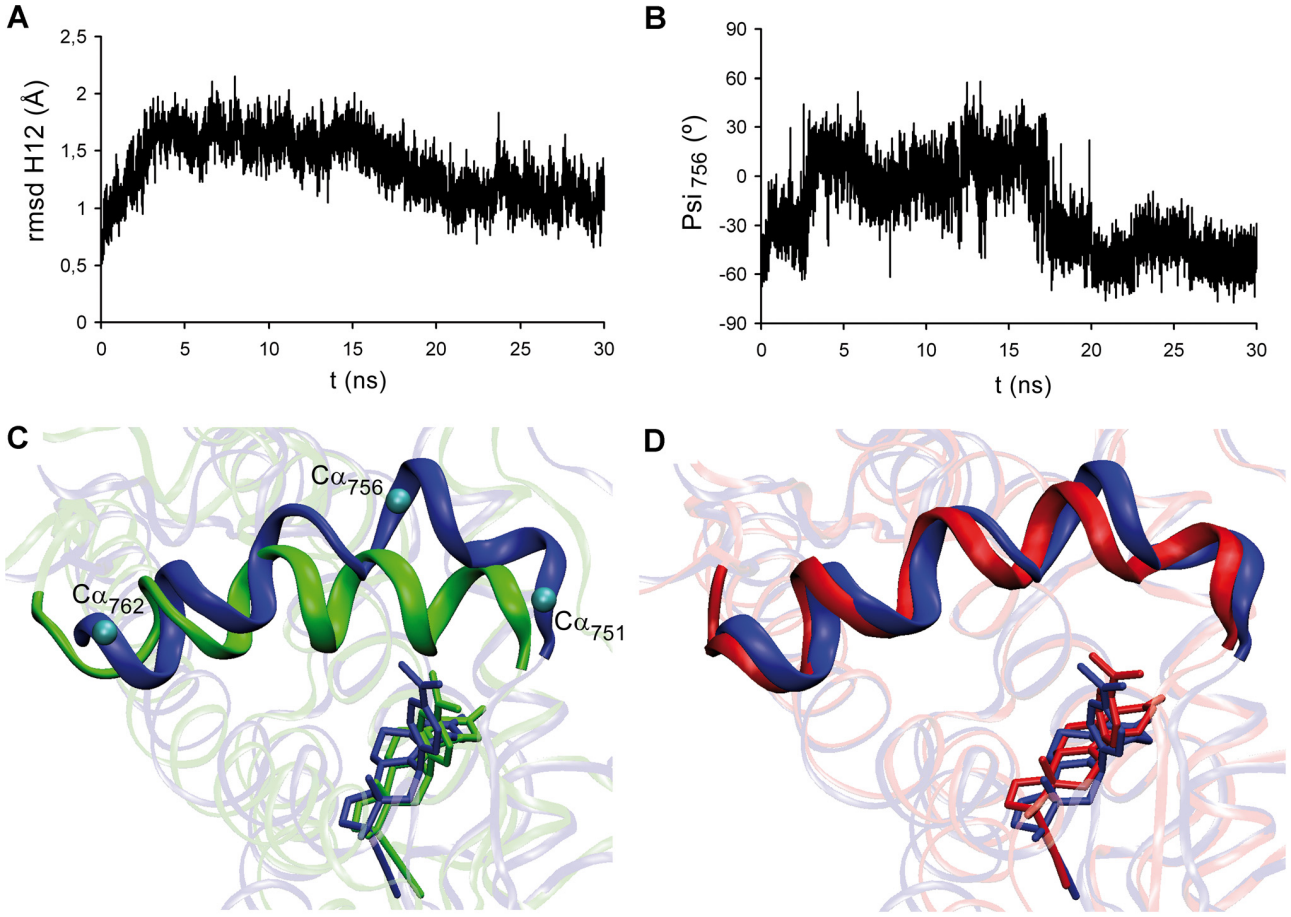
MD simulation of GR LBD-RU486 complex. **A**. Root mean squared deviation (rmsd) from the initial structure measured over the H12 (751–766) backbone atoms of the GR LBD-RU486 complex. **B**. Time evolution of psi_756_ angle. **C**. Superposition of the initial structure (green) of GR LBD-RU486 complex with the structure at 15 ns (blue). **D**. Superposition of the structures at 15 ns (blue) and at 30 ns (red).

Starting from this structure we performed 30 ns MD simulations for the corresponding complex. The root mean squared deviation (rmsd) from the initial structures measured over the backbone atoms of GR LBD-RU486 complex reveals that during the first 20 ns the system undergoes important conformational changes, resulting in the expansion of the receptor to accommodate the RU486 voluminous molecule (Figure S3C). Visual inspection of the GR LBD-RU486 trajectory shows that the main conformational changes occur in H12. During the first 3.5 ns the rmsd values for all H12 backbone atoms increased abruptly compared to the initial structure (Figure 7A), indicating a rapid RU486-induced destabilization of this helix. After this fast change, until 15 ns the rmsd remained essentially stable (average value = 1.61 Å). However, from 15 ns to 20 ns another H12 conformational change occurred with an rmsd decrease. Finally, from 20 ns to 30 ns H12 acquires a new stable conformation (average rmsd = 1.12 Å). Particularly from 5 to 15 ns, residues surrounding Ile756 loose their helical motif. The time evolution of the psi_756_ angle confirms that during this period dihedral values are not compatible with a helical structure (Figure 7B). However, on the last 10 ns simulation psi_756_ returns to values compatible with a helical structure. Additionally, from 5 to 15 ns the H12 is displaced and partially distorted with respect to the original conformation (Figure 7C). The structural basis for this distortion would reside in the fact that in order to accommodate the bulky moiety of RU486, H12 moves away from the ligand and particularly, residues 756–757 loose the helical motif. Interestingly, C-terminal residues acquire a helix motif, similar to that observed previously in both GR LBD-rigid steroid complexes [47], [48]. By comparing the distorted structure with a representative structure from the last 10 ns (Figure 7D) we observed that although H12 remains around the same position, residues 756–757 recover their helical motif. Taken together, these results suggest a receptor flexibility that allows it to accommodate RU486 by unwinding H12 specific residues and then stabilizing the helical motif in a different conformation.

Finally, MD studies of receptor-coactivator interaction were completed by the simulation of GR LBD-RU486/TIF2 complex, starting from the same initial structure as the other GR LBD-ligand/TIF2 complexes. Two main populations were found: one with short distance values between GR Glu755 and TIF2 corresponding to the first half simulation period; and the other with a larger distance corresponding to the second half period (Figure 6B, brown). Therefore, results show that when GR binds RU486, Glu755 and TIF2 form transient hydrogen bonds. B-factor values of backbone atoms in the four complexes were then calculated (Figure S4). Again, results show that the two antagonist ligands induce a larger TIF2 mobility compared to the agonist ligands.

In summary, MD simulation predict that hydrogen bonds between Glu755 (H12) and TIF2 are rapidly lost when 21OH-6,19OP is bound, and this lack of interactions leads to an increased fluctuation of the TIF2 peptide in comparison with GR-agonist complexes. Thus, 21OH-6,19OP would favor TIF2 dissociation from GR-LBD, consistently with Co-IP results (Figure 5B). The MD simulations also show that the hemisuccinate moiety in 21HS-6,19OP reverses the loss of receptor-coactivator interaction, in agreement with the experimental results.

## Discussion

In the present study we attempted to gain a deeper insight into activity modulation of the GR. By using four steroid ligands with different glucocorticoid activities we analyzed the effects of ligand structure on the GR transcriptional behavior and consequently the cellular glucocorticoid response. A precise characterization of the molecular determinants involved in specific GR conformational changes will contribute to understand the different GR mechanisms of action and may improve the strategies to the rational design of new selective drugs.

By comparing the behavior of different complexes, we observed that they distribute differently into the nucleus. Previous published studies [63], [64] have shown that as GR-ligand complexes are formed and translocated into the nucleus, they form focal domains consisting of several receptor molecules. GR intranuclear distribution would then depend on affinity-based differences between ligands rather than on transcriptional activities [50]. Here, we confirmed that high affinity ligands such as Dex or RU486 induce a highly punctuate distribution. On the contrary, rigid analogs distribute homogeneously. Although GR affinity of these steroids has not yet been determined, previous transactivation assays suggest that 21OH-6,19OP is a low affinity ligand, since only high concentrations ([10 µM]) are able to block Dex ([10 nM]) activity [48]. On the other hand, the apparent highest CV value of GR-21HS-6,19OP respect to the GR-21OH-6,19OP could reside in the increased ligand affinity due to the presence of the hemisuccinate moiety.

The ability of GR to homodimerize upon ligand binding was originally proposed based on several *in vitro* studies [25]–[29], [31]–[33], but leaving some unanswered key questions regarding its mechanism. Only recently *in vivo* dimerization of the receptor was visualized directly by co-IPs [39] or FRET analysis [40], and also through an elegant indirect experiment [36]. In this work, we used for the first time the *N&B* technique to evaluate GR dimerization *in vivo*. Interestingly, results indicate that most of GR molecules form homodimers inside the nucleus. Consistently, dimer formation was also visualized by *fluorescence fluctuation spectroscopy* in Cos-1 cells transfected with RXR-LBD-GFP fusion protein, obtaining similar results [65]. Furthermore, the GR oligomerization process would be independent of intranuclear distribution, since different GR distribution patterns showed similar oligomerization states.

For a variety of transcription factors, the route of DNA-protein assembly has been described by two main mechanisms: the *dimer pathway*, where transcription factors interact to DNA as homodimers; and the *monomer pathway* where two monomers bind DNA sequentially and assemble their dimerization interface while bound to DNA [66], [67]. Particularly on GR, it is still a debate issue whether GR homodimers are formed before or as a result of GRE binding. In this sense, some studies supported the *monomer pathway* hypothesis [30], [31], [33] while other studies provided evidences of GRE-independent dimer formation [25]–[29], [39], [40], [68]. In the *N&B* assays there is a large amount of GFPGR molecules due to overexpression, thus it is unlikely that enough active GREs at a given time point would be accessible to bind all GR molecules. Although we can not establish whether GR binds DNA as a dimer and/or as a monomer, results presented here support the idea that GR could form homodimers *in vivo* in a DNA-independent fashion. In agreement, Savory et al. suggested the possibility of GR homodimerization occurring during or even before the translocation process [36]. Furthermore, taking into account that homodimer formation is feasible even among non-functional complexes, according to our results the dimerization step would not be sufficient to define the GR as an active transcription factor.

MD simulation results led us to propose that the GR-21OH-6,19OP complex would be unable to homodimerize [47]. However, experimental studies performed here indicate that GR-21OH-6,19OP is able to form homocomplexes inside the nucleus of living cells. In this sense, considering functional assays which demonstrate that 21OH-6,19OP is unable to induce direct transactivation but it induces monomer transcriptional activities, we do not discard the possibility that dimers generated upon 21OH-6,19OP binding could acquire a conformation unable to fully activate GR-gene activating properties. Since the ability to induce transcription depends, at least in part, on the ligand-induced interaction with coactivators, one might postulate that the reduced transcriptional activation by a dissociated compound should indicate an impaired recruitment of coactivators. However, this hypothesis has not been investigated for most of the GR ligands [69]. In this sense, Co-IP assays demonstrated that GR-21OH-6,19OP is unable to recruit TIF2, explaining at least in part the molecular mechanism of action for this selective glucocorticoid. Therefore, in the presence of 21OH-6,19OP it is possible that GR AF-2 domain may adopt a conformation unable to bind TIF2 or that GR-21OH-6,19OP homocomplex may be blocked due to the binding to other proteins that impede cofactor recruitment. According to EMSAs results, this conformation would not affect the capacity of GR to bind to specific GREs, as all GR-ligand complexes are able to bind MMTV probe *in vitro*. In addition, the fact that GR-21OH-6,19OP complex is able to dimerize without recruiting TIF2 coactivator suggests that GR homodimers could be formed independently of cofactor interaction.

As mentioned above, agonist-complexes place Helix 12 in a permissive conformation able to bind TIF2 coactivator. In this sense, we observed that GR-TIF2 complex precipitates *in vivo* upon Dex or 21HS-6,19OP addition and both GR-agonist complexes increase their transactivation activities upon TIF2 overexpression. Although MD simulations allow us to investigate the molecular basis of dissociation rather than association mechanisms, results are consistent with the above experimental data since stable interactions between TIF2 peptide and GR LBD-agonist complexes were observed. On the other hand, MD simulation showed that hydrogen bonds between Glu755 (H12) and TIF2 are rapidly lost when 21OH-6,19OP is bound. In consequence, this lack of interaction leads to an increase fluctuation of the TIF2 peptide in comparison with the other GR-ligand complexes. Consistently, previous studies had shown that E755A and E755R mutations strongly compromise GR-Dex to recruit coactivators and hence to induce transactivation [12].

The MD study of GR LBD-RU486 complex showed that the presence of RU486 is sterically compatible with an H12 agonist position. These results may explain some agonistic actions of RU486 described elsewhere [20]. As it was observed in the PR LBD-RU486 complex [24], significant H12 conformational changes of GR LBD occur in order to accommodate the bulky moiety of RU486. This agrees with the idea of a dynamic model in which RU486 does not induce one particular receptor conformation, but works through changing H12 dynamic equilibrium. Accordingly, Co-IPs results indicate that GR-RU486 complex is able to interact with TIF2. Thus, it is possible that this ligand might generate a non-functional GR/TIF2 complex or that its antagonistic effect would reside on its ability to modulate the dynamics of GR-DNA interaction as previously suggested [60] and confirmed here. We conclude that 21OH-6,19OP and RU486 antagonize GR by different mechanisms. These results could also imply that while TIF2 recruitment may be necessary for GR transactivation activity at least on the MMTV model [70], [71], it appears not to be sufficient.

In view of the variety of physiological processes in which glucocorticoids are involved, from a pharmacological standpoint selective antiglucocorticoids that can block only some of these processes would be highly desirable. Interestingly, transactivation and transrepression studies suggest that 21OH-6,19OP conserves the so-called beneficial glucocorticoid properties while behaving as an antiglucocorticoid in unwanted actions. Nevertheless, increasing evidence suggests that glucocorticoid action could be classified in terms of GR-interacting coregulators rather than transactivation/dimer or transrepression/monomer activities. It is a currently accepted theory that both the identity and relative expression of coactivators and corepressors influence the ability of ligands to regulate gene expression [72]. Therefore, selective recruitment of specific coactivator subsets by determined GR conformations acquired upon ligand binding is likely to represent a promising goal into the design of new selective glucocorticoids.

## Materials and Methods

### Steroids and reagents

21OH-6,19OP and 21HS-6,19OP were prepared as previously described [73]. Dex and RU486 were purchased from Sigma and Dulbecco's modified Eagle's medium (DMEM) was from Invitrogen. Fetal calf serum (FCS) was purchased from Internegocios S.A. FCS was delipidated with charcoal-dextran as previously described [74].

### Cell culture and transient transfection assays

Cos-7, L929 and BHK21 cells were cultured in DMEM supplemented with 10% FCS plus penicillin (100 IU/ml) and streptomycin (100 µg/ml) at 37 C under humidified atmosphere with 4.5% CO_2_. Transient transfections were performed with Lipofectin 2000 (Invitrogen) according to manufacturer's instructions. After transfection, cells were incubated in DMEM containing 5% charcoal-stripped FCS and the corresponding steroids from 1000-fold stock solutions as indicated in each experiment.

### Transactivation and transrepression assays

3×10^4^ BHK cells were co-transfected with 0.3 µg pMMTV-luciferase vector [75] and 0.3 µg pTIF2 [76] or equal amounts of non-coding vector. For NFκB and AP-1 transrepression assays, 0.3 µg pkB-luciferase [77] and 0.1 µg pRelA vectors [78], or 0.3 µg pAP1- luciferase [79] and 0.1 µg pcJun [80] vectors were used. 0.2 µg pCMV-LacZ was added as control. Luciferase activity was measured according to the manufacturer's protocol (Promega Inc. cat # E1501). â-galactosidase activity was measured as previously described [81].

### Subcellular localization, intranuclear distribution and N&B analysis

3×10^5^ cells were transfected with 1 µg of pEGFPGR [49] or the empty vector pEGFP-C3 (Clontech) and incubated with the corresponding steroids for at least 40 minutes. Next, the medium was replaced with RAB buffer (Hepes 10 mM pH 7.4; NaCl 135 mM, KCl 10 mM, MgCl_2_ 0.4 mM; CaCl_2_ 1 mM; Glucose 1%) supplemented with the indicated steroids and then analyzed by confocal fluorescence microscopy. All measurements were done in a FV1000 confocal laser scanning microscope (Olympus), with an Olympus UPlanSApo 60× oil immersion objective (NA = 1.35). The excitation source was a multi-line Ar laser tuned at 488 nm (average power at the sample, 700 nW). Fluorescence was detected with a photomultiplier set in the pseudo photon-counting detection mode.

For intranuclear analysis, images of 512×512 pixels (pixel size 0.1 µm; pixel dwell time 20 µs) were taken, except for the representative pictures shown in Figure 1 (1024×1024 pixels). Coefficient of variation (CV) was calculated as described elsewhere [50]. The higher the CV value, the more nonrandom the distribution is. In each treatment, at least 20 cells were randomly selected and their CV values were averaged.

*N&B* measurements were done as previously described [53], [82] with some modifications. Briefly, for each studied cell a stack of 200 images (256×256 pixels) were taken in the conditions mentioned above, setting the pixel size to 82 nm and the pixel dwell time to 10 µs. Each stack was further analyzed using the *N&B* routine of the “GLOBALS for Images” program developed at the Laboratory for Fluorescence Dynamics (UCI, Irvine, CA). In this routine, the average fluorescence intensity (〈k〉) and its variance (σ^2^) at each pixel of an image are determined from the intensity values obtained at the given pixel along the images stack. The apparent brightness (B) is then calculated as the ratio of σ^2^ to 〈k〉 while the apparent number of moving particles (N) corresponds to the ratio of 〈k〉 to B. In a previous work it has been demonstrated that B is equal to the real brightness ε of the particles plus 1 [53]. Therefore, ε at every pixel of images can be easily extracted from B measurements. Importantly, this analysis only provides information regarding to the moving or fluctuating fluorescent molecules since fixed molecules will give B values equal to 1. Figure S5 shows an example of the analysis followed to determine the average brightness of GFPGR at each subcellular compartment.

### Electro mobility shift assay (EMSA)

EMSAs were performed with the previously described 33-bp MMTV GRE oligonucleotide probe [28]. The complementary strands were annealed in equimolar amounts (100 nM each) on 10 mM Tris-HCl pH 8; 1 mM EDTA; 30 mM KCl by denaturating at 95 C for 10 min and cooling down to room temperature. Double-stranded oligonucleotides were radiolabeled with T4 polynucleotide kinase (Invitrogen) and 50 µCi [γ^32^P] ATP. Proteins were extracted according to previous studies [83]. For binding studies, reactions were carried out in 25 µl reaction buffer (5 mM Tris-HCl, pH 8, 0.5 mM EDTA, 5% glycerol, 0.5 mM 2-mercaptoethanol, 1 µg poly(dI-dC), 90 ng Calf thymus DNA, and 30 µg BSA), 10 ng of radiolabeled probe and 200-fold excess of unlabeled specific or unspecific probe when indicated. Three micrograms of the nuclear extract were added to the binding reaction and incubated for 20 min at room temperature. Then, reaction mixtures were subjected to 6% acrylamide gel in Tris-Borate-EDTA solution. For dissociation studies, reactions were carried out in 100 µl reaction buffer containing 50 ng of radiolabeled probe and 18 µg of nuclear extract. After 20 minutes incubation at room temperature, 200-fold unlabeled probe was added and reaction aliquots (16 µl) were loaded at different times into a gel running at 200 V. Images were taken with STORM 820 PhosphorImager and analyzed with NIH-Image J v1.63 software analysis.

### Coimmunoprecipitation and western blot

2×10^6^ BHK cells co-transfected with 5 µg pRSV-hGR [84] and 5 µg pTIF2 were incubated with the corresponding steroids for 90 min at 37 C. Then, cells were lysed with CytoBuster Protein Extraction Buffer (EMD Biosciences) supplemented with Protease inhibitor cocktail (Calbiochem) and clarified by centrifugation at 13000 rpm for 5 min. Protein extracts (1 mg per treatment) were pre-cleared with protein A/G plus agarose solution (Santa Cruz Biotechnology; sc-2003). Samples were mixed by rotation for 1 h at 4 C and centrifuged for 2 min at 13000 rpm. Supernatants were immunoprecipitated with TIF2 antibody (Santa Cruz Biotechnology, sc-8996) and protein A/G plus agarose solution. Samples were then mixed by rotation for 4 h at 4 C and centrifuged for 3 min at 13000 rpm. Pellets were washed three times with TEGM buffer (10 mM HEPES pH 7.5, 1 mM EDTA, 20 mM Na_2_MoO_4_, 5% glycerol, 50 mM NaCl) and centrifuged for 3 min at 13000 rpm. Proteins were extracted with SDS sample buffer, separated by 7% SDS-PAGE and transferred to PVDF membrane (Bio-Rad) by electroblotting. Immunodetection was achieved with a 1∶1 mixture of GR antibodies (Santa Cruz Biotechnology, sc-1002; sc-1003).

### Coimmunoprecipitation in the presence of Ethidium bromide

Cells were lysed with CytoBuster Protein Extraction Buffer as described above. Then, Ethidium Bromide (100 µg/ml) was added to 200 µl of lysed material, followed by incubation for 1 h on ice. Then, after 5 min centrifugation at 13,000 rpm supernatants were collected and used for TIF2-GR immunoprecipitation as described above, with the exception that the immunoprecipitated material was then washed three times with TEGM buffer supplemented with Ethidium Bromide (100 µg/ml).

### Quantum Mechanics Calculations

The geometry of the ligands Dex, 21OH-6,19OP, 21HS-6,19OP and RU486 were optimized using the *ab initio* quantum chemistry program Gaussian 03 [85] and the HF/6-31G** basis set. RESP (restraint electrostatic potential) atomic charges were derived for all ligands using the optimized geometries.

### *In silico* construction of GR LBD-ligand/TIF2 complexes

The GR LBD-ligand/TIF2 complexes were built starting from the GR LBD-Dex complex (pdb:1 m2z) crystal structure using both the Chain A (corresponding to the receptor) and the Chain B (residues 759 to 773 corresponding to the TIF2 peptide). 21OH-6,19OP and 21HS-6,19OP were introduced within the GR LBD/TIF2 complex superimposing carbon atoms of ring C with the corresponding atoms of the Dex molecule in the GR LBD-Dex/TIF2 complex. The RU486 molecule was introduced similarly to the GR LBD-RU486 complex described below.

### *In silico* construction of GR LBD-RU486 complex

To introduce the RU486 within the GR LBD, the chain A of the crystal GR LBD-Dex complex (pdb:1 m2z) was superimposed with the crystal structure of the GR LBD-RU486 complex (pdb:1 nhz) using the VMD program [86]. Then, the crystalized RU486 molecule was replaced for the *optimized* RU486 structure by superimposing the corresponding carbon atoms of the C ring. These RU486 coordinates and GR LBD pdb:1 m2z coordinates were used to construct the complex. When the RU486 molecule is introduced in this way in the GR-LDB, 11-substituent diethyl amino group atoms and Leu753 (H12) side chain atoms overlap giving rise to sterical clashes (Figure S3B). To resolve these clashes Leu753 side chain was rotated using the Deep-view/Swiss-pdbviewer program [87] until Leu753 (orange) side chain acquired a similar conformation as the corresponding residue (Met909) of the PR LBD-RU486 complex [24] (Figure S3A). Final accommodation of RU486 diethyl amino group and Leu753 side chain was achieved by geometry optimization.

### Molecular Dynamics Simulations

MD simulations were performed by using the AMBER 9 software package [88]. The complexes were immersed in an octahedral box of TIP3P water molecules using the Leap module. The Amber99 force field parameters were used for all protein residues [89] and ligand parameters were assigned with the general AMBER force field (GAFF) and the corresponding RESP charges using the Antechamber module of AMBER. The systems were initially optimized and then gradually heated to 300 K. Starting from these equilibrated structures, MD production runs of 30 ns for GR LBD-RU486 complex and 20 ns for the GR LBD-ligand/TIF2 complexes were performed. All simulations were performed at 1 atm and 300 K, maintained with the Berendsen barostat and thermostat [90] using periodic boundary conditions and the particle mesh Ewald method (grid spacing of 1 Å) for treating long-range electrostatic interactions, with a uniform neutralizing plasma. The SHAKE algorithm was used to keep bonds involving H atoms at their equilibrium length, allowing us to employ a 2 fs time step for the integration of Newton's equations. The analysis of the trajectories was performed with the Ptraj module of Amber and the visualization of the structures with the VMD program [86].

### Statistical Analysis

Results were expressed as means ± standard error. Statistical analyses were performed with STATISTICA 6.0 (StatSoft, Inc.) and consisted of one- or two-way ANOVA followed by Tukey's multiple comparisons tests. Differences were regarded as significant at *P*<0.05. Before statistical analysis, data were tested for normality and homoscedasticity using Lilliefors and Bartlett's tests, respectively. In some cases, log-transformed or roothsquare-transformed data were used. In all cases, bars with different superscript letters are significantly different from each other.

## Supporting Information

Figure S1 Ligand effect on GFPGR transcriptional activity and nuclear translocation. A. Transactivation assays. BHK21 cells were cotransfected with pEGFPGR and MMTV-Luc reporter vector. pCMV-LacZ vector were also introduced. Cells were incubated for 18 h with the indicated steroids combination at the following final concentrations: ethanol and/or DMSO (Control), 10 nM Dexamethasone (Dex), 10 μM 21-Hydroxy-6,19-epoxyprogesterone (21OH-6,19OP), 10 μM 21-succinoyloxy-6,19-epoxyprogesterone (21HS-6,19OP), and 1 μM mifepristone (RU486). Luciferase activity was measured. After correcting for β-galactosidase activity, values were expressed as fold induction relative to the control. Means ± S.E. from three independent experiments are shown. ANOVA test were not performed because homoscedasticity could not be achieved. Instead, a t-student test was carried out only between two pairs of treatments. Thus, bars with different superscript letters (a vs. b and c vs. d) are significantly different from each other (*P* <0.05). B. Cellular localization of GFPGR molecules. Cos-7, L929, and BHK21 cells transfected with pEGFPGR were incubated with the indicated steroids for 40 min at 37°C as described in “Materials and Methods”. Cells were visualized by confocal scanning microscopy. Scale bar  =  20 μm. The figure shows representative cells for each treatment. In the upper left side of dex treated L929 cells it shows GFP transfected cells showing homogeneous distribution throughout the cell. Note that the GR-complex does not seem to translocate completely in the presence of 21OH-6,19OP.
(3.37 MB TIF)

Figure S2 Stability of GR-ligands - TIF2 complexes during the 30 ns simulation. Root mean squared deviation (rmsd) from the initial structure measured over the backbone atoms of the GR LBD-dex/TIF2 (red), GR LBD-21HS-6,19OP/TIF2 (green) (A), or GR LBD-21OH-6,19OP/TIF2 (blue) and GR LBD-RU486/TIF2 (brown) (B).
(0.95 MB TIF)

Figure S3 *In silico* introduction of RU486 into the GR LBD and stability of the complex during simulation. A. In the PR LBD-RU486 crystal structure (pdb:2w8y), the RU486 diethyl amino group occupy the space between Met909 and H3. B. When the RU486 molecule is introduced *in silico* in the GR-LDB, 11-substituent diethyl amino group atoms (Cyan: carbon; Red: Oxygen; Blue: Nitrogen) and Leu753 (H12) side chain atoms (green) overlap giving rise to sterical clashes. To resolve these clashes Leu753 side chain was rotated using the Deep-view/Swiss-pdbviewer program [87] until Leu753 (orange) side chain acquired a similar conformation as the corresponding residue (Met909) of the PR LBD-RU486 complex [24]. Final accommodation of RU486 diethyl amino group and Leu753 side chain was achieved by geometry optimization. C. Root mean squared deviation (rmsd) from the initial structure measured over the backbone atoms of the GR LBD-RU486 complex.
(5.54 MB TIF)

Figure S4 TIF2 fluctuation within the GR LBD-ligand complexes. B-factor of the TIF2 backbone atoms in the GR LBD-Dex/TIF2 (red), GR LBD-21HS-6,19OP/TIF2 (green), GR LBD-21OH-6,19OP/TIF2 (blue) and GR LBD-RU486/TIF2 (brown) complexes.
(0.42 MB TIF)

Figure S5 Measurement of GFPGR molecule brightness. A. Picture of a representative cell treated with 21OH-6,19OP. B. As described in “Materials and Methods,” the average fluorescence intensity and its variance at each pixel of an image are determined from the intensity values obtained at the given pixel along the images stack. The apparent brightness (B) is then calculated as the ratio of the average fluorescence intensity and its variance. For stimulated cells, the fluorescence intensity at the nucleus was higher than the intensity at the cytosol. Therefore we applied an intensity threshold to calculate separately the average value of B in both cell regions. For unstimulated cells, B values were calculated in squared regions which only included points of the cytoplasm or the nucleus. The figure shows the B values histogram for two regions of a representative stimulated-cell. The left-shifted histogram (blue) corresponds to the cytoplasmic region (red spots, left cell box). The right-shifted histogram (black) corresponds to the nucleus (red spots, right cell box). The mean of each Gaussian-fit histogram is the B value for each cell compartment. Note that B values from the nucleus are in average higher than cytoplasmic values. This indicates a higher oligomerization state in the nucleus respect to the cytoplasm. Finally, ε (real brightness) is B minus 1 [53].
(2.20 MB TIF)

## References

[1] Gross KL, Cidlowski JA (2008). Tissue-specific glucocorticoid action: a family affair.. Trends Endocrinol Metab.

[2] van der Laan S, Meijer OC (2008). Pharmacology of glucocorticoids: beyond receptors.. Eur J Pharmacol.

[3] McMaster A, Ray DW (2007). Modelling the glucocorticoid receptor and producing therapeutic agents with anti-inflammatory effects but reduced side-effects.. Exp Physiol.

[4] Kleiman A, Tuckermann JP (2007). Glucocorticoid receptor action in beneficial and side effects of steroid therapy: lessons from conditional knockout mice.. Mol Cell Endocrinol.

[5] Necela BM, Cidlowski JA (2004). Mechanisms of glucocorticoid receptor action in noninflammatory and inflammatory cells.. Proc Am Thorac Soc.

[6] Chen T (2008). Nuclear receptor drug discovery.. Curr Opin Chem Biol.

[7] Olivier Kassel PH (2007). Crosstalk between the glucocorticoid receptor and other transcription factors: Molecular aspects.. Molecular and Cellular Endocrinology.

[8] Newton R, Holden NS (2007). Separating transrepression and transactivation: a distressing divorce for the glucocorticoid receptor?. Mol Pharmacol.

[9] Clark AR (2007). Anti-inflammatory functions of glucocorticoid-induced genes.. Mol Cell Endocrinol.

[10] Kumar R, Thompson EB (2005). Gene regulation by the glucocorticoid receptor: structure: function relationship.. J Steroid Biochem Mol Biol.

[11] Gronemeyer H, Gustafsson JA, Laudet V (2004). Principles for modulation of the nuclear receptor superfamily.. Nat Rev Drug Discov.

[12] Bledsoe RK, Montana VG, Stanley TB, Delves CJ, Apolito CJ (2002). Crystal structure of the glucocorticoid receptor ligand binding domain reveals a novel mode of receptor dimerization and coactivator recognition.. Cell.

[13] Kauppi B, Jakob C, Farnegardh M, Yang J, Ahola H (2003). The three-dimensional structures of antagonistic and agonistic forms of the glucocorticoid receptor ligand-binding domain: RU-486 induces a transconformation that leads to active antagonism.. J Biol Chem.

[14] Madauss KP, Bledsoe RK, McLay I, Stewart EL, Uings IJ (2008). The first X-ray crystal structure of the glucocorticoid receptor bound to a non-steroidal agonist.. Bioorg Med Chem Lett.

[15] Suino-Powell K, Xu Y, Zhang C, Tao YG, Tolbert WD (2008). Doubling the size of the glucocorticoid receptor ligand binding pocket by deacylcortivazol.. Mol Cell Biol.

[16] Biggadike K, Bledsoe RK, Hassell AM, Kirk BE, McLay IM (2008). X-ray crystal structure of the novel enhanced-affinity glucocorticoid agonist fluticasone furoate in the glucocorticoid receptor-ligand binding domain.. J Med Chem.

[17] Veleiro AS, Alvarez LD, Eduardo SL, Burton G (2010). Structure of the glucocorticoid receptor, a flexible protein that can adapt to different ligands.. Chem Med Chem.

[18] Stahn C, Lowenberg M, Hommes DW, Buttgereit F (2007). Molecular mechanisms of glucocorticoid action and selective glucocorticoid receptor agonists.. Mol Cell Endocrinol.

[19] Frego L, Davidson W (2006). Conformational changes of the glucocorticoid receptor ligand binding domain induced by ligand and cofactor binding, and the location of cofactor binding sites determined by hydrogen/deuterium exchange mass spectrometry.. Protein Sci.

[20] Schulz M, Eggert M, Baniahmad A, Dostert A, Heinzel T (2002). RU486-induced glucocorticoid receptor agonism is controlled by the receptor N terminus and by corepressor binding.. J Biol Chem.

[21] Wang Q, Blackford JA, Song LN, Huang Y, Cho S (2004). Equilibrium interactions of corepressors and coactivators with agonist and antagonist complexes of glucocorticoid receptors.. Mol Endocrinol.

[22] He Y, Szapary D, Simons SS (2002). Modulation of induction properties of glucocorticoid receptor-agonist and -antagonist complexes by coactivators involves binding to receptors but is independent of ability of coactivators to augment transactivation.. J Biol Chem.

[23] Schoch GA, D'Arcy B, Stihle M, Burger D, Bar D Molecular switch in the glucocorticoid receptor: active and passive antagonist conformations.. J Mol Biol.

[24] Raaijmakers HC, Versteegh JE, Uitdehaag JC (2009). The X-ray structure of RU486 bound to the progesterone receptor in a destabilized agonistic conformation.. J Biol Chem.

[25] Wrange O, Eriksson P, Perlmann T (1989). The purified activated glucocorticoid receptor is a homodimer.. J Biol Chem.

[26] Chalepakis G, Schauer M, Cao XA, Beato M (1990). Efficient binding of glucocorticoid receptor to its responsive element requires a dimer and DNA flanking sequences.. DNA Cell Biol.

[27] Cairns W, Cairns C, Pongratz I, Poellinger L, Okret S (1991). Assembly of a glucocorticoid receptor complex prior to DNA binding enhances its specific interaction with a glucocorticoid response element.. J Biol Chem.

[28] Drouin J, Sun YL, Tremblay S, Lavender P, Schmidt TJ (1992). Homodimer formation is rate-limiting for high affinity DNA binding by glucocorticoid receptor.. Mol Endocrinol.

[29] Segard-Maurel I, Rajkowski K, Jibard N, Schweizer-Groyer G, Baulieu EE (1996). Glucocorticosteroid receptor dimerization investigated by analysis of receptor binding to glucocorticosteroid responsive elements using a monomer-dimer equilibrium model.. Biochemistry.

[30] Liu W, Wang J, Yu G, Pearce D (1996). Steroid receptor transcriptional synergy is potentiated by disruption of the DNA-binding domain dimer interface.. Mol Endocrinol.

[31] Tsai SY, Carlstedt-Duke J, Weigel NL, Dahlman K, Gustafsson JA (1988). Molecular interactions of steroid hormone receptor with its enhancer element: evidence for receptor dimer formation.. Cell.

[32] Dahlman-Wright K, Siltala-Roos H, Carlstedt-Duke J, Gustafsson JA (1990). Protein-protein interactions facilitate DNA binding by the glucocorticoid receptor DNA-binding domain.. J Biol Chem.

[33] Dahlman-Wright K, Wright A, Gustafsson JA, Carlstedt-Duke J (1991). Interaction of the glucocorticoid receptor DNA-binding domain with DNA as a dimer is mediated by a short segment of five amino acids.. J Biol Chem.

[34] Heck S, Kullmann M, Gast A, Ponta H, Rahmsdorf HJ (1994). A distinct modulating domain in glucocorticoid receptor monomers in the repression of activity of the transcription factor AP-1.. EMBO J.

[35] Reichardt HM, Kaestner KH, Tuckermann J, Kretz O, Wessely O (1998). DNA binding of the glucocorticoid receptor is not essential for survival.. Cell.

[36] Savory JG, Prefontaine GG, Lamprecht C, Liao M, Walther RF (2001). Glucocorticoid receptor homodimers and glucocorticoid-mineralocorticoid receptor heterodimers form in the cytoplasm through alternative dimerization interfaces.. Mol Cell Biol.

[37] Adams M, Meijer OC, Wang J, Bhargava A, Pearce D (2003). Homodimerization of the glucocorticoid receptor is not essential for response element binding: activation of the phenylethanolamine N-methyltransferase gene by dimerization-defective mutants.. Mol Endocrinol.

[38] Mikuni S, Tamura M, Kinjo M (2007). Analysis of intranuclear binding process of glucocorticoid receptor using fluorescence correlation spectroscopy.. FEBS Lett.

[39] Dewint P, Gossye V, De Bosscher K, Vanden Berghe W, Van Beneden K (2008). A plant-derived ligand favoring monomeric glucocorticoid receptor conformation with impaired transactivation potential attenuates collagen-induced arthritis.. J Immunol.

[40] Roberson S, Allie-Reid F, Vanden Berghe W, Visser K, Binder A (2009). Abrogation of glucocorticoid receptor dimerization correlates with dissociated glucocorticoid behavior of compound A.. J Biol Chem.

[41] Nagaich AK, Rayasam GV, Martinez ED, Becker M, Qiu Y (2004). Subnuclear trafficking and gene targeting by steroid receptors.. Ann N Y Acad Sci.

[42] Hager GL, Elbi C, Johnson TA, Voss T, Nagaich AK (2006). Chromatin dynamics and the evolution of alternate promoter states.. Chromosome Res.

[43] Ogawa H, Yu RT, Haraguchi T, Hiraoka Y, Nakatani Y (2004). Nuclear structure-associated TIF2 recruits glucocorticoid receptor and its target DNA.. Biochem Biophys Res Commun.

[44] Voss TC, John S, Hager GL (2006). Single-cell analysis of glucocorticoid receptor action reveals that stochastic post-chromatin association mechanisms regulate ligand-specific transcription.. Mol Endocrinol.

[45] Vicent GP, Monteserin MC, Veleiro AS, Burton G, Lantos CP (1997). 21-Hydroxy-6,19-oxidoprogesterone: a novel synthetic steroid with specific antiglucocorticoid properties in the rat.. Mol Pharmacol.

[46] Veleiro AS, Nevado MV, Monteserin MC, Burton G (1995). Syntheses of 21-hydroxy-11,19-oxidopregn-4-ene-3, 20-dione and 21-hydroxy- 6,19-oxidopregn-4-ene-3, 20-dione.. Steroids.

[47] Alvarez LD, Marti MA, Veleiro AS, Presman DM, Estrin DA (2008). Exploring the molecular basis of action of the passive antiglucocorticoid 21-hydroxy-6,19-epoxyprogesterone.. J Med Chem.

[48] Alvarez LD, Marti MA, Veleiro AS, Misico RI, Estrin DA (2008). Hemisuccinate of 21-hydroxy-6,19-epoxyprogesterone: a tissue-specific modulator of the glucocorticoid receptor.. ChemMedChem.

[49] Galigniana MD, Scruggs JL, Herrington J, Welsh MJ, Carter-Su C (1998). Heat shock protein 90-dependent (geldanamycin-inhibited) movement of the glucocorticoid receptor through the cytoplasm to the nucleus requires intact cytoskeleton.. Mol Endocrinol.

[50] Schaaf MJ, Lewis-Tuffin LJ, Cidlowski JA (2005). Ligand-selective targeting of the glucocorticoid receptor to nuclear subdomains is associated with decreased receptor mobility.. Mol Endocrinol.

[51] Htun H, Holth LT, Walker D, Davie JR, Hager GL (1999). Direct visualization of the human estrogen receptor alpha reveals a role for ligand in the nuclear distribution of the receptor.. Mol Biol Cell.

[52] Veleiro AS, Pecci A, Monteserin MC, Baggio R, Garland MT (2005). 6,19-Sulfur-bridged progesterone analogues with antiimmunosuppressive activity.. J Med Chem.

[53] Digman MA, Dalal R, Horwitz AF, Gratton E (2008). Mapping the number of molecules and brightness in the laser scanning microscope.. Biophys J.

[54] Digman MA, Wiseman PW, Choi C, Horwitz AR, Gratton E (2009). Stoichiometry of molecular complexes at adhesions in living cells.. Proc Natl Acad Sci U S A.

[55] Walker D, Htun H, Hager GL (1999). Using inducible vectors to study intracellular trafficking of GFP-tagged steroid/nuclear receptors in living cells.. Methods.

[56] Rusconi S, Yamamoto KR (1987). Functional dissection of the hormone and DNA binding activities of the glucocorticoid receptor.. EMBO J.

[57] Aumais JP, Lee HS, DeGannes C, Horsford J, White JH (1996). Function of directly repeated half-sites as response elements for steroid hormone receptors.. J Biol Chem.

[58] Guido EC, Delorme EO, Clemm DL, Stein RB, Rosen J (1996). Determinants of promoter-specific activity by glucocorticoid receptor.. Mol Endocrinol.

[59] Groyer A, Schweizer-Groyer G, Cadepond F, Mariller M, Baulieu EE (1987). Antiglucocorticosteroid effects suggest why steroid hormone is required for receptors to bind DNA in vivo but not in vitro.. Nature.

[60] Pandit S, Geissler W, Harris G, Sitlani A (2002). Allosteric effects of dexamethasone and RU486 on glucocorticoid receptor-DNA interactions.. J Biol Chem.

[61] Willmann T, Beato M (1986). Steroid-free glucocorticoid receptor binds specifically to mouse mammary tumour virus DNA.. Nature.

[62] Lai JS, Herr W (1992). Ethidium bromide provides a simple tool for identifying genuine DNA-independent protein associations.. Proc Natl Acad Sci U S A.

[63] van Steensel B, Brink M, van der Meulen K, van Binnendijk EP, Wansink DG (1995). Localization of the glucocorticoid receptor in discrete clusters in the cell nucleus.. J Cell Sci.

[64] Htun H, Barsony J, Renyi I, Gould DL, Hager GL (1996). Visualization of glucocorticoid receptor translocation and intranuclear organization in living cells with a green fluorescent protein chimera.. Proc Natl Acad Sci U S A.

[65] Chen Y, Wei LN, Muller JD (2003). Probing protein oligomerization in living cells with fluorescence fluctuation spectroscopy.. Proc Natl Acad Sci U S A.

[66] Kim B, Little JW (1992). Dimerization of a specific DNA-binding protein on the DNA.. Science.

[67] Kohler JJ, Metallo SJ, Schneider TL, Schepartz A (1999). DNA specificity enhanced by sequential binding of protein monomers.. Proc Natl Acad Sci U S A.

[68] Eriksson P, Wrange O (1990). Protein-protein contacts in the glucocorticoid receptor homodimer influence its DNA binding properties.. J Biol Chem.

[69] Kassel O, Herrlich P (2007). Crosstalk between the glucocorticoid receptor and other transcription factors: molecular aspects.. Mol Cell Endocrinol.

[70] Bhandare R, Damera G, Banerjee A, Flammer JR, Keslacy S Glucocorticoid receptor interacting protein-1 restores glucocorticoid responsiveness in steroid-resistant airway structural cells.. Am J Respir Cell Mol Biol.

[71] Li X, Wong J, Tsai SY, Tsai MJ, O'Malley BW (2003). Progesterone and glucocorticoid receptors recruit distinct coactivator complexes and promote distinct patterns of local chromatin modification.. Mol Cell Biol.

[72] Smith CL, O'Malley BW (2004). Coregulator function: a key to understanding tissue specificity of selective receptor modulators.. Endocr Rev.

[73] Burton G, Lantos C, Veleiro A (2006). Method for the preparation of 21-hydroxy-6,19-oxidoprogesterone (21OH-6,19OP)..

[74] Lippman M, Bolan G, Huff K (1976). The effects of androgens and antiandrogens on hormone-responsive human breast cancer in long-term tissue culture.. Cancer Res.

[75] Horwitz KB, Zava DT, Thilagar AK, Jensen EM, McGuire WL (1978). Steroid receptor analyses of nine human breast cancer cell lines.. Cancer Res.

[76] Nojek IM, Werbajh SE, Colo GP, Rubio FM, Franco LD (2004). [Different enzymatic activities recruitment by specific domains of TIF2 are involved in NF-kappaB transactivation].. Medicina (B Aires).

[77] Costas MA, Muller Igaz L, Holsboer F, Arzt E (2000). Transrepression of NF-kappaB is not required for glucocorticoid-mediated protection of TNF-alpha-induced apoptosis on fibroblasts.. Biochim Biophys Acta.

[78] Molinero LL, Fuertes MB, Girart MV, Fainboim L, Rabinovich GA (2004). NF-kappa B regulates expression of the MHC class I-related chain A gene in activated T lymphocytes.. J Immunol.

[79] Marinissen MJ, Chiariello M, Tanos T, Bernard O, Narumiya S (2004). The small GTP-binding protein RhoA regulates c-jun by a ROCK-JNK signaling axis.. Mol Cell.

[80] Iavarone C, Catania A, Marinissen MJ, Visconti R, Acunzo M (2003). The platelet-derived growth factor controls c-myc expression through a JNK- and AP-1-dependent signaling pathway.. J Biol Chem.

[81] Truss M, Bartsch J, Schelbert A, Hache RJ, Beato M (1995). Hormone induces binding of receptors and transcription factors to a rearranged nucleosome on the MMTV promoter in vivo.. Embo J.

[82] Dalal RB, Digman MA, Horwitz AF, Vetri V, Gratton E (2008). Determination of particle number and brightness using a laser scanning confocal microscope operating in the analog mode.. Microsc Res Tech.

[83] Andrews NC, Faller DV (1991). A rapid micropreparation technique for extraction of DNA-binding proteins from limiting numbers of mammalian cells.. Nucleic Acids Res.

[84] Godowski PJ, Rusconi S, Miesfeld R, Yamamoto KR (1987). Glucocorticoid receptor mutants that are constitutive activators of transcriptional enhancement..

[85] Gaussian 03 RC, Frisch MJ (2004).

[86] Humphrey W, Dalke A, Schulten K (1996). VMD: visual molecular dynamics.. J Mol Graph.

[87] Guex N, Peitsch MC (1997). SWISS-MODEL and the Swiss-PdbViewer: an environment for comparative protein modeling.. Electrophoresis.

[88] Pearlman DA, Case DA, Caldwell JW, Ross WS, Cheatham TE (1995). AMBER, a package of computer programs for applying molecular mechanics, normal mode analysis, molecular dynamics and free energy calculations to simulate the structural and energetic properties of molecules.. Comput Phys Commun.

[89] Cheatham TE, Cieplak P, Kollman PA (1999). A modified version of the Cornell et al. force field with improved sugar pucker phases and helical repeat.. Biomolecular Structure and Dynamics.

[90] Berendsen HJC, Postma JPM, Van Gunsteren WF, DiNola A, Haak JR (1984). Molecular dynamics with coupling to an external bath.. J Chem Phys.

[91] Lu NZ, Cidlowski JA (2005). Translational regulatory mechanisms generate N-terminal glucocorticoid receptor isoforms with unique transcriptional target genes.. Mol Cell.

