# Supplementary figures and images for: Insights on Glucocorticoid Receptor Activity Modulation through the Binding of Rigid Steroids
Source: PLoS One. 2010 Oct 11;5(10):e13279. doi: 10.1371/journal.pone.0013279 (PMC2952596; doi:10.1371/journal.pone.0013279)

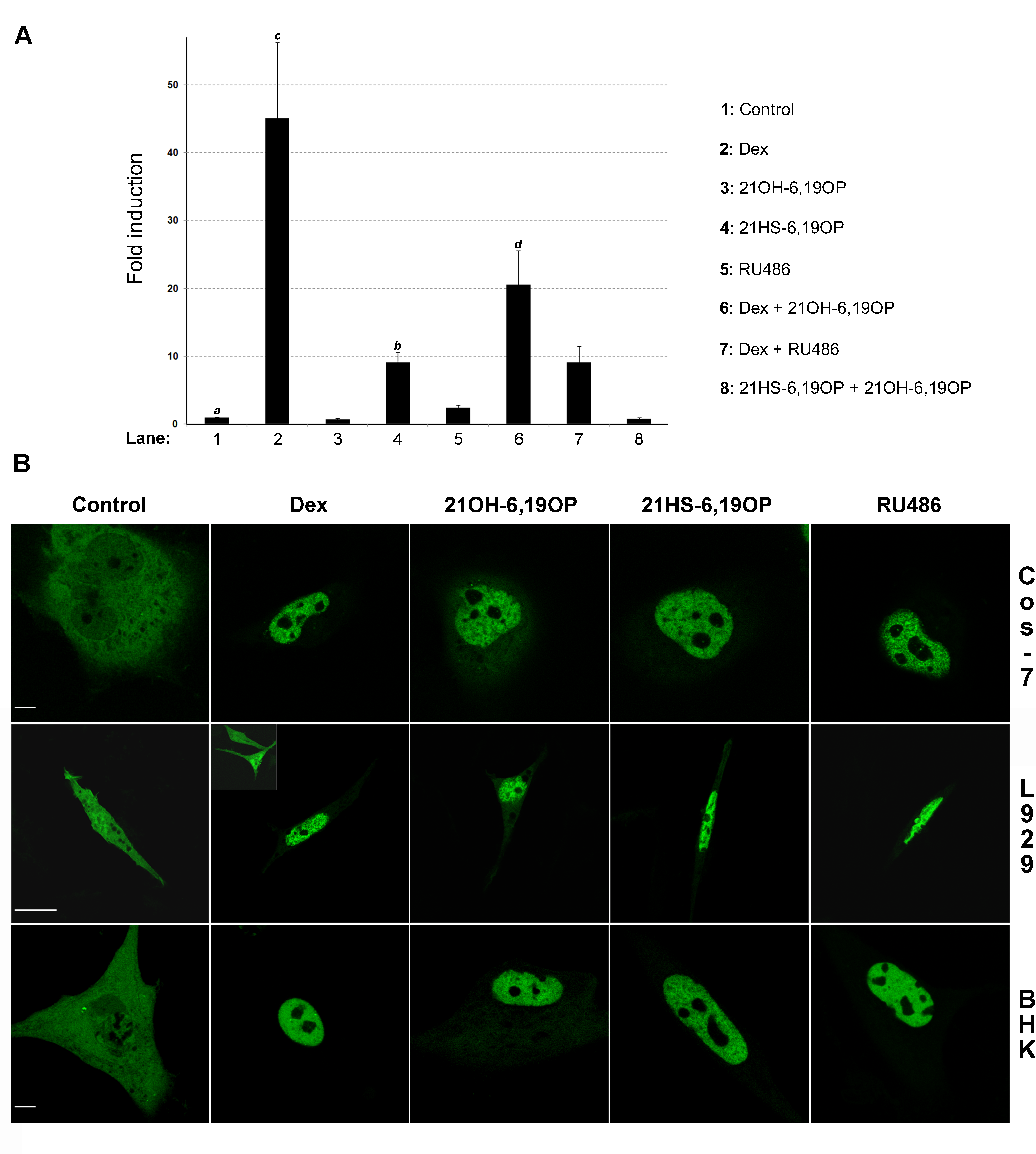

Supplement: Figure S1 — Ligand effect on GFPGR transcriptional activity and nuclear translocation. A. Transactivation assays. BHK21 cells were cotransfected with pEGFPGR and MMTV-Luc reporter vector. pCMV-LacZ vector were also introduced. Cells were incubated for 18 h with the indicated steroids combination at the following final concentrations: ethanol and/or DMSO (Control), 10 nM Dexamethasone (Dex), 10 μM 21-Hydroxy-6,19-epoxyprogesterone (21OH-6,19OP), 10 μM 21-succinoyloxy-6,19-epoxyprogesterone (21HS-6,19OP), and 1 μM mifepristone (RU486). Luciferase activity was measured. After correcting for β-galactosidase activity, values were expressed as fold induction relative to the control. Means ± S.E. from three independent experiments are shown. ANOVA test were not performed because homoscedasticity could not be achieved. Instead, a t-student test was carried out only between two pairs of treatments. Thus, bars with different superscript letters (a vs. b and c vs. d) are significantly different from each other (P <0.05). B. Cellular localization of GFPGR molecules. Cos-7, L929, and BHK21 cells transfected with pEGFPGR were incubated with the indicated steroids for 40 min at 37°C as described in “Materials and Methods”. Cells were visualized by confocal scanning microscopy. Scale bar = 20 μm. The figure shows representative cells for each treatment. In the upper left side of dex treated L929 cells it shows GFP transfected cells showing homogeneous distribution throughout the cell. Note that the GR-complex does not seem to translocate completely in the presence of 21OH-6,19OP. (3.37 MB TIF) [file pone.0013279.s001.tif]

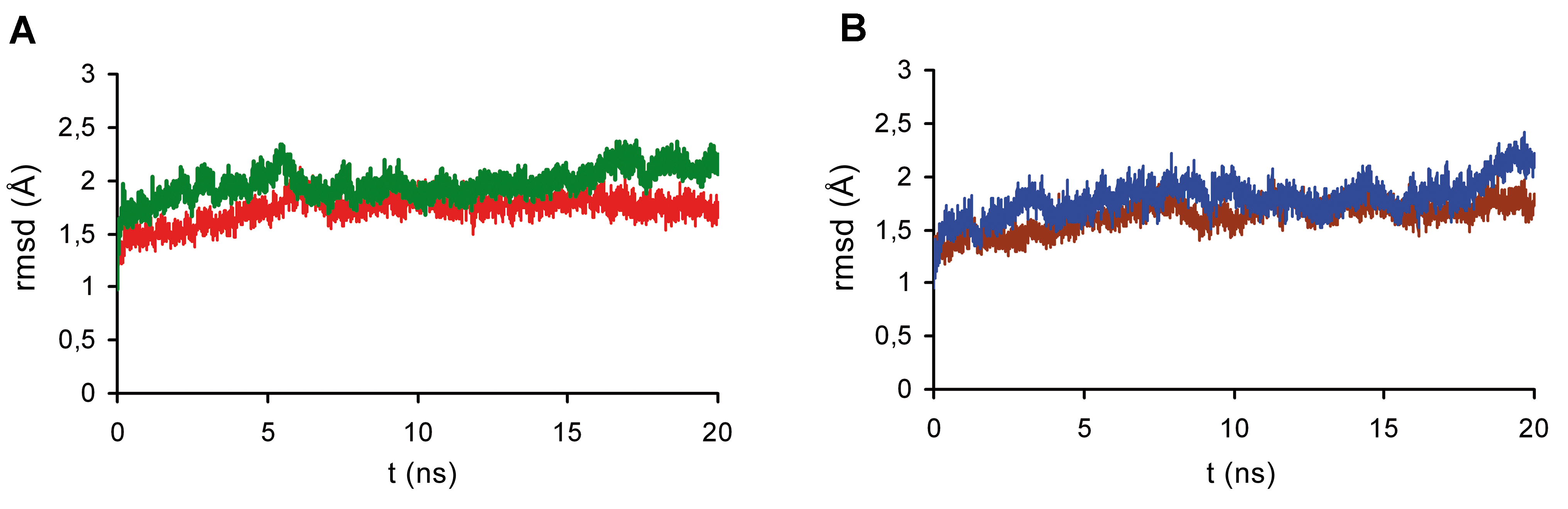

Supplement: Figure S2 — Stability of GR-ligands - TIF2 complexes during the 30 ns simulation. Root mean squared deviation (rmsd) from the initial structure measured over the backbone atoms of the GR LBD-dex/TIF2 (red), GR LBD-21HS-6,19OP/TIF2 (green) (A), or GR LBD-21OH-6,19OP/TIF2 (blue) and GR LBD-RU486/TIF2 (brown) (B). (0.95 MB TIF) [file pone.0013279.s002.tif]

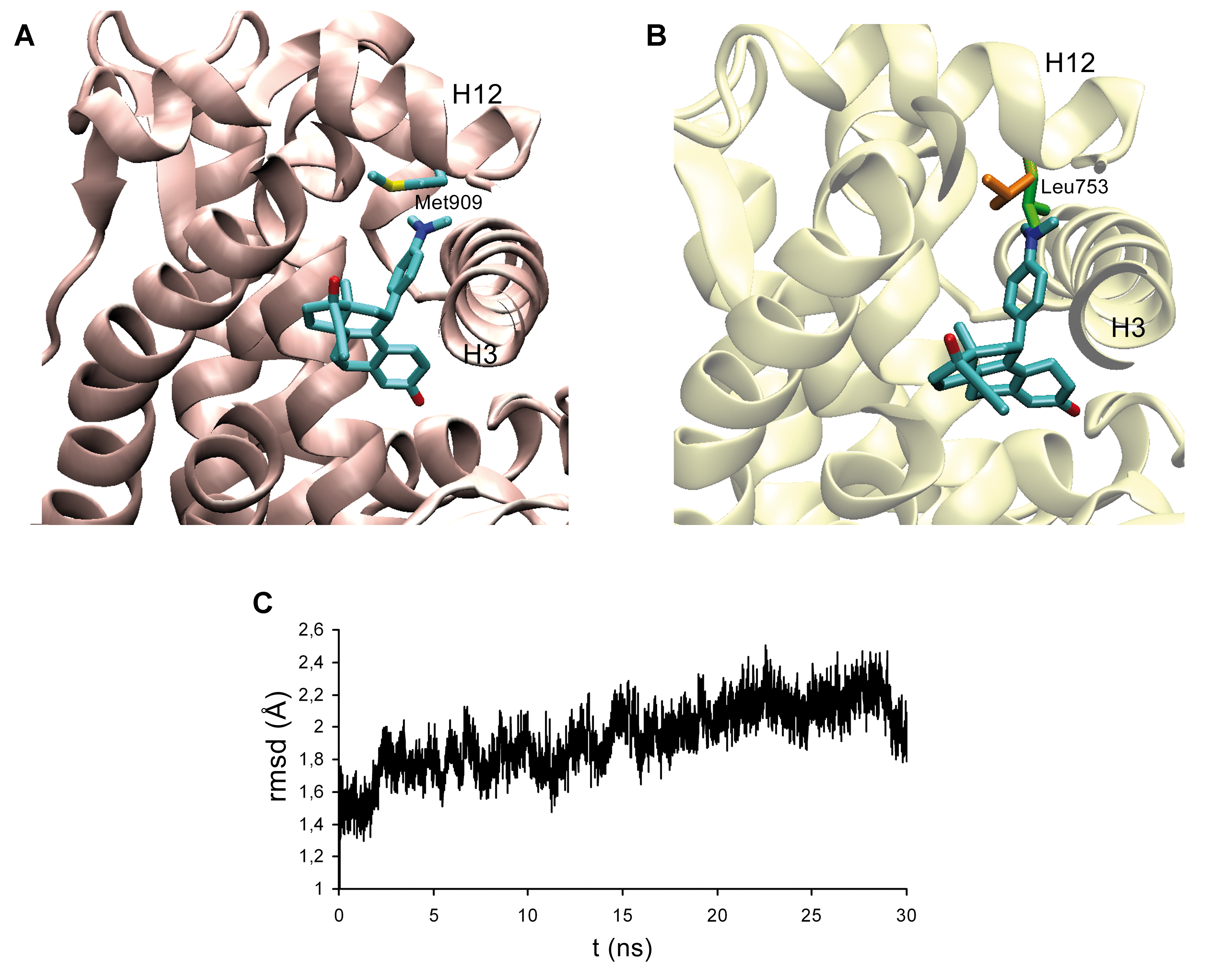

Supplement: Figure S3 — In silico introduction of RU486 into the GR LBD and stability of the complex during simulation. A. In the PR LBD-RU486 crystal structure (pdb:2w8y), the RU486 diethyl amino group occupy the space between Met909 and H3. B. When the RU486 molecule is introduced in silico in the GR-LDB, 11-substituent diethyl amino group atoms (Cyan: carbon; Red: Oxygen; Blue: Nitrogen) and Leu753 (H12) side chain atoms (green) overlap giving rise to sterical clashes. To resolve these clashes Leu753 side chain was rotated using the Deep-view/Swiss-pdbviewer program [87] until Leu753 (orange) side chain acquired a similar conformation as the corresponding residue (Met909) of the PR LBD-RU486 complex [24]. Final accommodation of RU486 diethyl amino group and Leu753 side chain was achieved by geometry optimization. C. Root mean squared deviation (rmsd) from the initial structure measured over the backbone atoms of the GR LBD-RU486 complex. (5.54 MB TIF) [file pone.0013279.s003.tif]

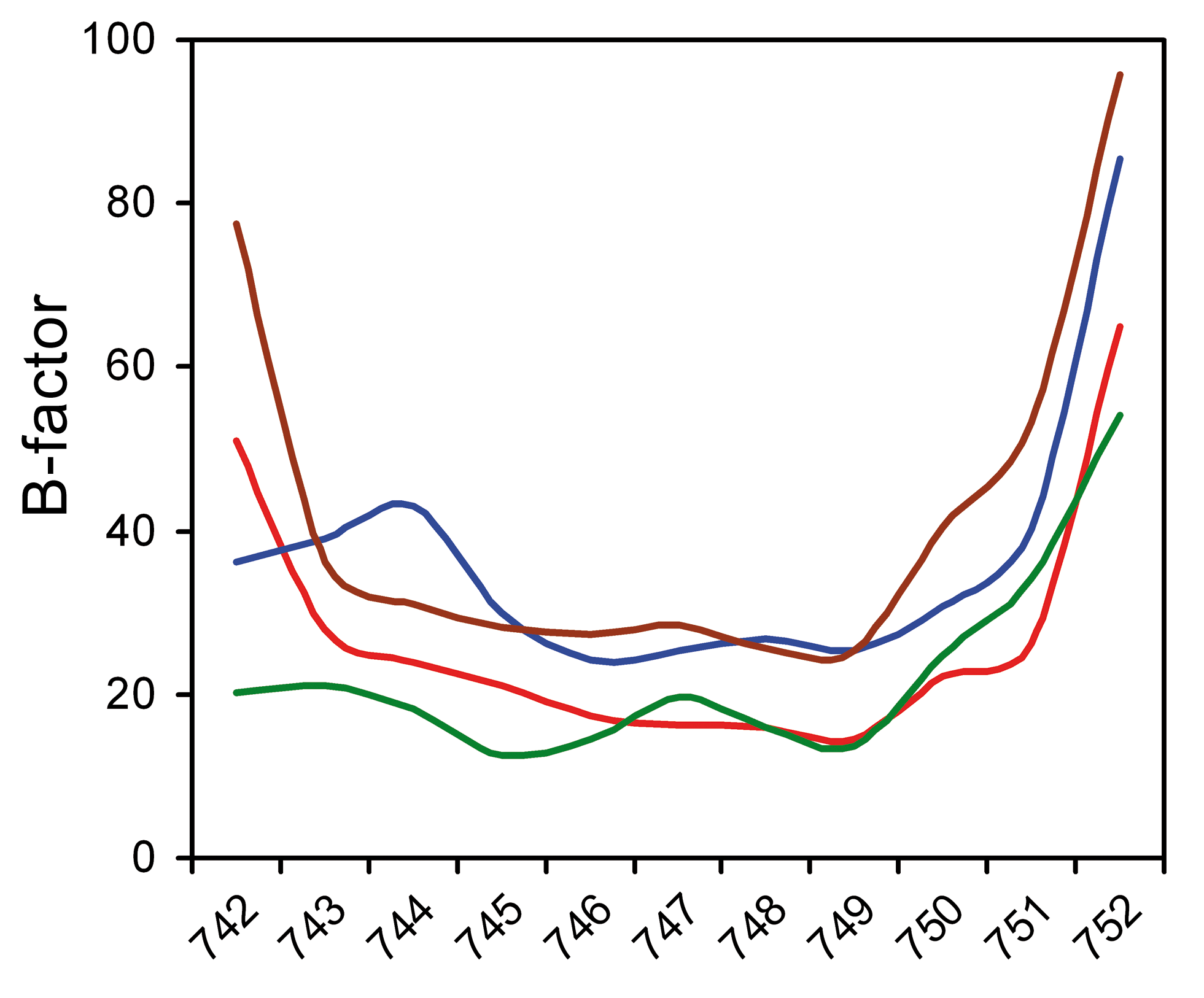

Supplement: Figure S4 — TIF2 fluctuation within the GR LBD-ligand complexes. B-factor of the TIF2 backbone atoms in the GR LBD-Dex/TIF2 (red), GR LBD-21HS-6,19OP/TIF2 (green), GR LBD-21OH-6,19OP/TIF2 (blue) and GR LBD-RU486/TIF2 (brown) complexes. (0.42 MB TIF) [file pone.0013279.s004.tif]

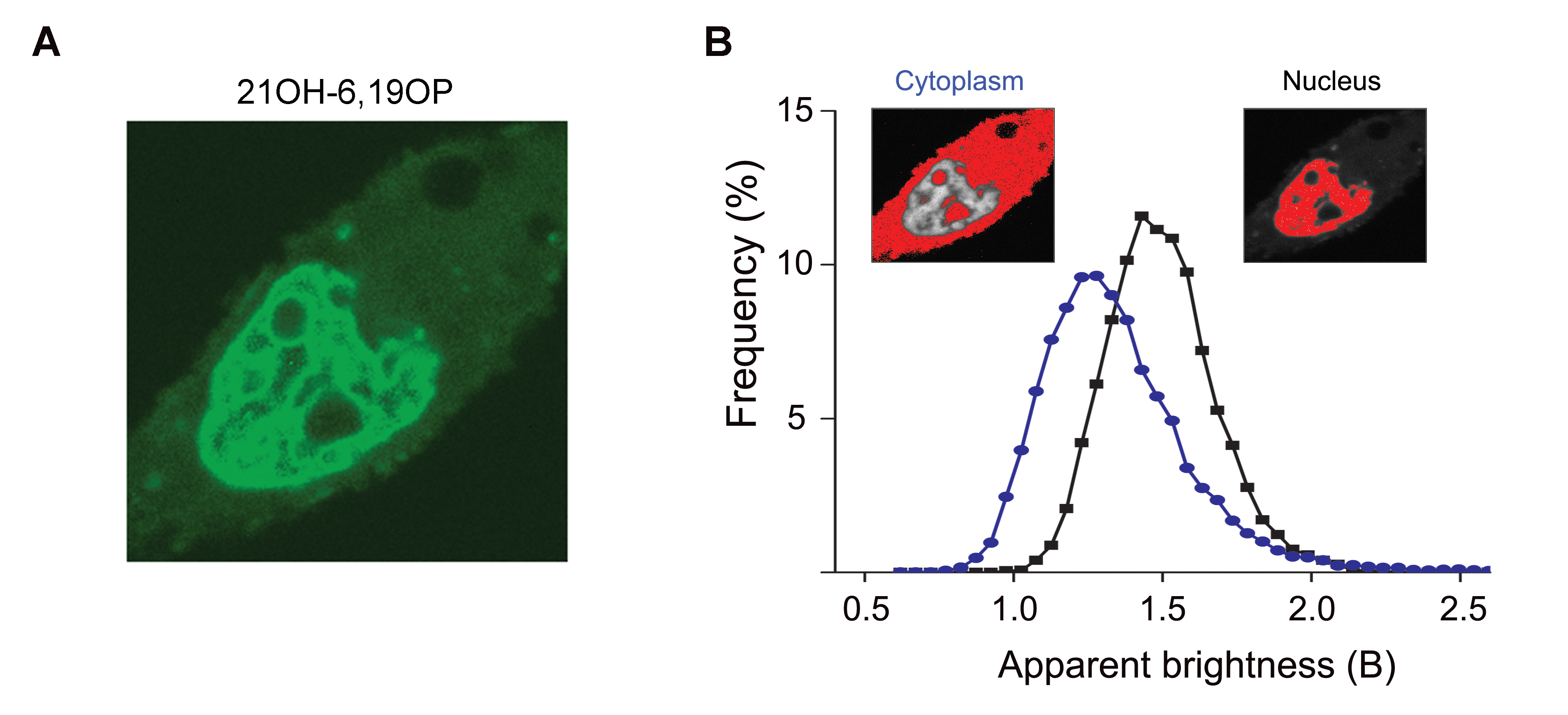

Supplement: Figure S5 — Measurement of GFPGR molecule brightness. A. Picture of a representative cell treated with 21OH-6,19OP. B. As described in “Materials and Methods,” the average fluorescence intensity and its variance at each pixel of an image are determined from the intensity values obtained at the given pixel along the images stack. The apparent brightness (B) is then calculated as the ratio of the average fluorescence intensity and its variance. For stimulated cells, the fluorescence intensity at the nucleus was higher than the intensity at the cytosol. Therefore we applied an intensity threshold to calculate separately the average value of B in both cell regions. For unstimulated cells, B values were calculated in squared regions which only included points of the cytoplasm or the nucleus. The figure shows the B values histogram for two regions of a representative stimulated-cell. The left-shifted histogram (blue) corresponds to the cytoplasmic region (red spots, left cell box). The right-shifted histogram (black) corresponds to the nucleus (red spots, right cell box). The mean of each Gaussian-fit histogram is the B value for each cell compartment. Note that B values from the nucleus are in average higher than cytoplasmic values. This indicates a higher oligomerization state in the nucleus respect to the cytoplasm. Finally, ε (real brightness) is B minus 1 [53]. (2.20 MB TIF) [file pone.0013279.s005.tif]
